# Combined Toxicity of Microplastics and Antimicrobials on Animals: A Review

**DOI:** 10.3390/antibiotics14090896

**Published:** 2025-09-05

**Authors:** Kuok Ho Daniel Tang

**Affiliations:** Department of Environmental Science, The University of Arizona, Tucson, AZ 86721, USA; danielkhtang@arizona.edu

**Keywords:** antibiotics, antifungals, antivirals, co-exposure, ecotoxicity, microplastics, plastisphere

## Abstract

**Background/Objectives**: Microplastics are ubiquitous pollutants that pose physical toxicity and serve as vectors for antimicrobial agents, altering their bioavailability and toxicity. Unlike previous reviews that focus solely on antibiotics and terrestrial or aquatic ecosystems, this review integrates recent findings on the combined impacts of microplastics and antimicrobials on both aquatic and terrestrial animals, highlighting their biological responses. **Methods**: Recent experimental studies involving aquatic and terrestrial animals published in peer-reviewed journals were reviewed. These studies employed co-exposure designs using microplastics of different sizes, aging conditions, and surface chemistries in combination with antimicrobial compounds. **Results**: Microplastics combined with antimicrobials cause species-specific and often synergistic toxicity in aquatic organisms, affecting reproduction, immunity, oxidative stress, gene expression, and microbiota, with co-exposure often amplifying adverse physiological and developmental effects. Similarly, co-exposure to microplastics and antimicrobials in rodents, amphibians, birds, and soil invertebrates frequently leads to synergistic toxicity, oxidative stress, disrupted gut microbiota, and enhanced accumulation and bioavailability of pollutants, promoting inflammation, neurotoxicity, metabolic dysfunction, and increased antibiotic resistance gene propagation. Particle size, aging, and antimicrobial type influence toxicity severity. Certain microplastic-antimicrobial combinations can exhibit antagonistic effects, though less frequently reported. **Conclusions**: The interactions between microplastics and antimicrobials pose heightened risks to the health of organisms and ecological stability. These findings underscore the need to revise current risk assessment protocols to consider pollutant mixtures and microplastics-mediated transport. Future research should focus on environmentally relevant exposures, mechanistic studies using omics tools, and long-term ecological impacts. Integrated regulatory strategies are essential to address the compounded effects of microplastics and chemical contaminants.

## 1. Introduction

Since the 1950s, global plastic production has risen sharply, reaching approximately 460 million tons in 2024. This represents an increase from 435 million tons in 2020 and 400.3 million tons in 2022. Despite this rapid growth, less than 9% of all plastics produced have been recycled through mechanical or chemical processes [1]. The combination of surging production and low recycling rates has resulted in significant plastic accumulation in aquatic environments. Approximately 1 to 1.7 million tons of plastics enter the oceans each year, accounting for around 0.5% of global plastic waste [2,3]. These plastics degrade gradually under environmental stressors, such as ultraviolet radiation, wave action, and biological activity, forming microplastics—plastic fragments smaller than 5 mm [4].

The environmental prevalence of microplastics and their ingestion by organisms across different trophic levels are well-documented [5,6]. Once ingested, microplastics can transfer through food webs and interact with other pollutants due to their large surface area and hydrophobic nature, which enables them to adsorb toxic chemicals [7,8]. Both laboratory simulations and field observations have confirmed this pollutant-carrying behavior [9,10,11]. When consumed, these particles can deliver hazardous substances into organisms, a phenomenon known as the “Trojan horse” effect [12]. However, the actual level of pollutant accumulation depends on variables such as the reversibility of chemical sorption and the concentration gradients between microplastics and the exposed organisms [13,14].

Furthermore, simultaneous exposure to microplastics and environmental contaminants can interfere with biological detoxification processes, potentially leading to additive (a combined effect of two or more stressors that is equal to the sum of their individual effects, with no amplification or reduction), synergistic (combined exposure amplifies harm beyond what would be expected from each alone), or antagonistic (combined toxicity of two substances is less than the sum of their individual toxicities) toxic effects [15,16]. Combined exposure has been shown to increase oxidative stress and acute toxicity [17,18]. For instance, treatment with polystyrene and sulfamethoxazole led to significant mortality and developmental abnormalities across various developmental stages and anatomical regions in zebrafish [19]. Chloramphenicol and polystyrene microplastics, whether applied separately or together, inhibited algal growth, with the combined exposure resulting in greater toxicity than either substance alone [20]. Interestingly, in certain cases, such as the interaction between microplastics and roxithromycin in red tilapia, microplastics may mitigate specific toxic effects, including neurotoxicity [21]. Zhang et al. [22] also observed that, compared to exposure to roxithromycin alone, co-exposure with 1-μm polystyrene particles significantly decreased glutathione peroxidase and malondialdehyde levels in *Daphnia magna*, while co-exposure with 10-μm polystyrene notably reduced glutathione S-transferase and malondialdehyde responses.

**Figure 1:**
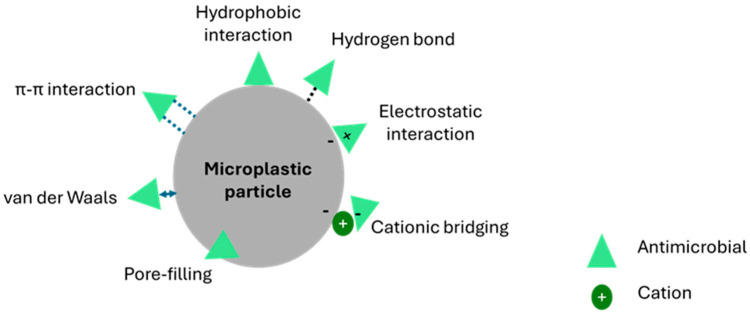
Interactions between microplastics and antimicrobials.

The co-occurrence of microplastics and antimicrobials in the environment raises urgent concerns about their combined ecological impacts [23]. Antimicrobials, which have significantly improved human and animal health and agricultural productivity, now frequently contaminate the environment due to sources like agricultural runoff and industrial discharge [24]. Physicochemically, microplastics interact with antimicrobials mainly through adsorption processes driven by hydrophobic interactions, electrostatic attraction/repulsion, hydrogen bonding, and π-π stacking (Figure 1). For instance, hydrophobic polymers like polyethylene or polystyrene readily adsorb non-polar antimicrobials, while charged or functionalized surfaces (from weathering or aging, i.e., the changes that microplastics undergo after being released into the environment) can bind ionizable antibiotics such as sulfonamides or tetracyclines [25]. Their environmental presence not only poses ecological risks but also contributes to the proliferation of resistance genes [26,27]. In 2021, estimates show 1.14 million deaths directly caused by antimicrobial resistance and 4.71 million deaths associated with antimicrobial resistance. Forecasts indicate that by 2050, annual deaths directly attributable to antimicrobial resistance could reach 1.91 million, with the total associated deaths possibly rising to 8.22 million globally [28]. Microplastics act as vectors for antibiotic resistance genes (ARGs) and pathogens by providing a stable and nutrient-rich surface, known as the plastisphere, for microbial colonization. The hydrophobicity and roughness of plastic surfaces promote biofilm formation, where high cell density and close contact facilitate horizontal gene transfer, including the exchange of ARGs [4]. Additionally, microplastics can adsorb antibiotics and heavy metals from the environment, creating selective pressures that favor resistant strains. Pathogenic bacteria such as *Vibrio* sp. and *Escherichia coli* have been detected on microplastics, raising concerns that these particles can disseminate both ARGs and harmful microbes through aquatic ecosystems and food webs [29,30]. For instance, Su et al. [31] found that in aquaculture, larger microplastics carried higher ARG loads, especially multidrug-resistant and high-risk (level I–II) genes, suggesting enhanced potential for gene transfer. They also concentrated pathogens like *Brucella* and *Pseudomonas*, underscoring risks to aquaculture safety and public health.

Emerging research indicates that microplastics may increase the toxicity of antimicrobials in animals [32], yet there is a lack of reviews that comprehensively present the combined toxicity. Zhang et al. [33] reviewed the combined toxicity of microplastics and antibiotics in aquatic environments. The review did not extend to terrestrial organisms and other antimicrobials. Similarly, the meta-analysis conducted by Yu et al. [34] on combined microplastic and antibiotic toxicity focuses only on aquatic ecosystems. Wang et al. [35] reviewed how microplastics interact with antibiotics in water bodies, with toxicity being a minor component of the review, which mainly covers adsorption mechanisms and their influencing factors. The review by Wang et al. [36] also emphasizes the adsorption and desorption of antibiotics on microplastics more than the combined toxic effects. While the review by Wei et al. [37] includes the soil environment, it primarily emphasizes environmental fate and interactions. Additionally, the extant reviews rarely include other antimicrobials, particularly antifungals [32,33,37].

To address this knowledge gap, the present review comprehensively presents the combined toxicity of microplastics and antimicrobials on animals in terrestrial and aquatic environments. By synthesizing experimental data from diverse studies, this review also aims to identify key drivers of toxicity variation. This review contributes to a valuable foundation for future studies on the combined toxicity of microplastics and antimicrobials. It also supports more accurate environmental risk assessments and policy development aimed at mitigating the impacts of these co-occurring contaminants.

## 2. Review Methodology

This review adopted a narrative approach, based on a comprehensive search of major scientific databases, including Web of Science, Scopus, and ScienceDirect for articles published from 2015 up to June 2025. These databases have been chosen because they are multidisciplinary and offer high-quality, peer-reviewed journals in environmental science and ecotoxicology, which align closely with the goals of this review. The following combinations of keywords were used:

- “microplastics” AND “antimicrobials” OR “antibiotics” OR “antifungals” OR “antivirals”
- “combined toxicity” OR “co-exposure” OR “joint effects”
- “toxicity” AND “animals” OR “aquatic organisms” OR “invertebrates” OR “fish”
- Specific antimicrobial names (e.g., sulfamethoxazole, tetracycline, ciprofloxacin) AND “microplastics”

**Figure 2:**
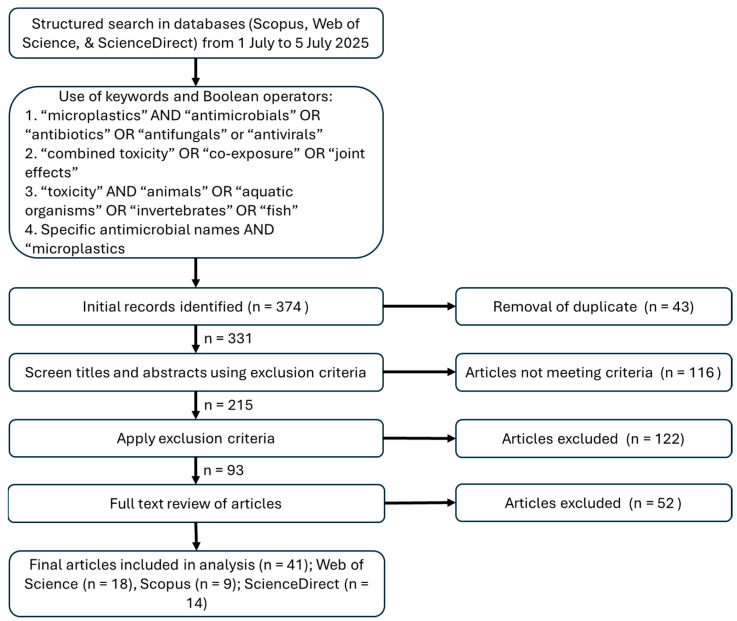
Flowchart showing the literature review process.

Only articles published in English were included. Studies were included if they: (1) investigated the combined (not individual) effects of microplastics and antibiotics; (2) used animal models (including aquatic and terrestrial invertebrates and vertebrates); and (3) reported toxicological outcomes such as mortality, growth inhibition, oxidative stress, immune response, behavioral effects, or histopathological changes. Exclusion criteria included: (1) studies focusing solely on individual pollutants; (2) reviews, editorials, or commentaries (though their references were screened for relevant primary studies); and (3) studies on microbial communities unless animal hosts were involved. Figure 2 shows the article selection process. The list of articles included for full-text review, along with the corresponding keywords or key terms used during the literature search, can be found in the Supplementary Materials.

It is worth noting that this review only uses three main databases for its literature search, given its environmental and ecotoxicological focus. ScienceDirect was included because of its particularly strong coverage in environmental sciences, engineering, materials science, energy, and health. Combining Scopus, Web of Science, and ScienceDirect enables triangulation to verify consistency in indexing, coverage, and citation data. It does not include databases like PubMed, which could provide valuable information, although it primarily focuses on biomedical and clinical topics. Additionally, it does not utilize Google Scholar, which includes both peer-reviewed (searchable through these databases) and non-peer-reviewed articles. The exclusion of PubMed and Google Scholar represents a significant limitation of this review. A quick search on Google Scholar revealed that most of the peer-reviewed papers included here match those found on Google Scholar.

## 3. Toxicity on Aquatic Animals

The widespread presence of microplastics and antimicrobials in aquatic environments has raised growing concern due to their potential to exert combined toxic effects on aquatic organisms. When aquatic animals are simultaneously exposed to both pollutants, the interactions between microplastics and antimicrobials can alter their bioavailability, toxicity, and ecological behavior [38,39,40]. This co-exposure may lead to enhanced bioaccumulation, oxidative stress, immune suppression, reproductive toxicity, and disruptions in gut microbiota, often exceeding the effects observed from either pollutant alone [34].

### 3.1. Crustaceans

**Figure 3:**
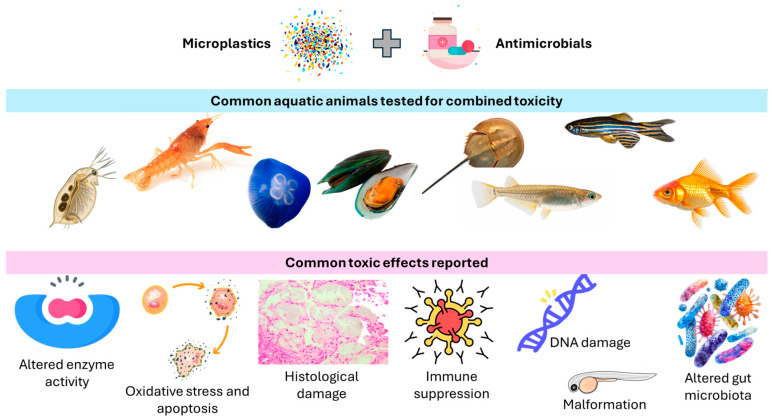
Common aquatic animal models used for studying the combined toxicity of microplastics and antimicrobials, and the common toxic effects reported.

Zhang et al. [22] examined the individual and combined effects of 1-μm and 10-μm polystyrene microplastics and the antibiotic roxithromycin on *Daphnia magna* (Figure 3) using acute and sublethal toxicity tests. The 48 h EC_50_ values were 66.97 mg/L for 1-μm polystyrene, 199.94 mg/L for 10-μm polystyrene, and 20.28 mg/L for roxithromycin. Oxidative stress was assessed via malondialdehyde levels and the activity of four enzymes: catalase, glutathione peroxidase, glutathione S-transferase, and superoxide dismutase (Table 1). Exposure to low concentrations of polystyrene microplastics (0.1 mg/L) or roxithromycin (0.01 mg/L) alone increased catalase and glutathione S-transferase activity and elevated malondialdehyde levels. However, co-exposure with 1-μm polystyrene reduced glutathione peroxidase activity and malondialdehyde levels compared to roxithromycin alone, while 10-μm polystyrene co-exposure lowered glutathione S-transferase activity and malondialdehyde levels [22].

Building on these findings, Liu et al. [41] examined the intergenerational effects of polystyrene microplastics and the antibiotic roxithromycin, alone and in combination, at environmentally relevant levels using *Daphnia magna* (Figure 3). Ultraviolet (UV)-aged polystyrene microplastics improved the survival of the parental generation (F0) exposed to roxithromycin by 20–40% at 0.1 and 10 µg/L. The negative impacts of roxithromycin on reproduction, including reduced offspring numbers and growth rate, were also lessened. However, in the first-generation offspring (F1), reproductive toxicity was more severe under both single and combined exposures, with combined treatments showing slower recovery. Co-exposure shifted the inhibitory effect of roxithromycin on swimming speed and acceleration to stimulation, though feeding remained suppressed. Acetylcholinesterase activity increased 1.61–3.25 times in all treatments, with UV-aged microplastics causing stronger effects than pristine ones. While single exposures to roxithromycin or polystyrene triggered antioxidant responses (total antioxidant capacity, superoxide dismutase), co-exposure led to a marked rise in oxidative damage, indicated by increased malondialdehyde levels [41] (Table 1).

Furthermore, Yin et al. [42] assessed both acute and chronic toxicity of 5.8 μm polystyrene microplastics and their interactions with three antimicrobials, i.e., triclosan, triclocarban, and methyl-triclosan, in *Daphnia magna*. Polystyrene exhibited low acute toxicity, with an EC_50_ (half maximum effective concentration) of 36.5 mg/L. At 1 mg/L, polystyrene did not significantly affect the acute toxicity of the antimicrobial agents. However, during a 21-day chronic exposure, polystyrene at concentrations ≥2 mg/L showed notable reproductive toxicity by delaying the first brood, reducing brood frequency, and lowering total offspring. When combined with the antimicrobials, polystyrene further intensified their adverse effects on reproduction. Methyl-triclosan, in the presence of polystyrene, led to the greatest decrease in population growth (Table 1) [42].

Nugnes et al. [43] examined the chronic and sub-chronic effects of 1.0 μm polystyrene microplastics, both alone and in combination with the antiviral drug acyclovir and the insecticide imidacloprid, on *Ceriodaphnia dubia*, a highly sensitive freshwater crustacean. In short-term (24 h) exposure assessed by the Comet assay, the mixtures generally showed antagonistic genotoxicity. However, during a 7-day exposure, the combinations produced mainly additive chronic toxicity at environmentally relevant concentrations, ranging from a few to tens of ng/L for polystyrene microplastics, low μg/L for acyclovir, and from low to hundreds of μg/L for imidacloprid. After 7 days of exposure, acyclovir and polystyrene microplastics showed the most pronounced chronic toxicity, with median concentrations causing reduced reproduction measured in the low microgram per liter range—hundredths of μg/L for acyclovir and single-digit μg/L for polystyrene microplastics [43]. In the chronic toxicity tests, the most substantial reproductive effect was observed with a 1:3 mixture of polystyrene microplastics and acyclovir, leading to a 54.8% decrease in offspring production at concentrations of 0.15 μg/L for polystyrene microplastics and 0.0379 μg/L for acyclovir (Table 1). These effects would typically require higher individual concentrations, approximately 2 μg/L for polystyrene microplastics and 0.8 μg/L for acyclovir, indicating a potentiation at lower doses in combination. In genotoxicity assessments, the effect of the combined exposure appeared to depend subtly on acyclovir concentration. When polystyrene microplastic levels were relatively high, they were the primary driver of toxicity. However, as acyclovir concentrations increased, the overall impact lessened, possibly due to interactions between acyclovir and polystyrene microplastics, such as acyclovir binding to the microplastics, which might shield their reactive sites and lower their bioavailability or toxicity [43].

Said et al. [44] examined how pyrogallol and microplastics, alone and together, affected *Procambarus clarkii* (Figure 3) over 15 days at concentrations of 10 mg/L pyrogallol and 100 mg/L microplastics. Both treatments, separately and combined, significantly altered total hemocyte counts (granular and semi-granular) versus controls. Co-exposure raised aspartate aminotransferase, alanine aminotransferase, and protein levels (Table 1). Single pyrogallol or microplastic exposures reduced superoxide dismutase, glutathione, and total antioxidant capacity while increasing catalase and malondialdehyde. Hepatopancreas histology revealed vacuolation, tubule degradation, eosinophilic deposits, hemocyte infiltration, and other structural abnormalities under all toxic treatments (Table 1).

### 3.2. Mollusks

As for the mollusks, Han et al. [45] examined the immunotoxicity of three antibiotics, namely oxytetracycline (270 ng/L), florfenicol (42 ng/L), and sulfamethoxazole (140 ng/L), with or without polystyrene microplastics (0.26 mg/L, 500 nm) in thick-shelled mussels (Figure 3). Results showed that all treatments impaired immune function, with combined exposure to antibiotics and microplastics causing stronger effects. Compared to antibiotics alone, co-exposure to oxytetracycline, florfenicol, and sulfamethoxazole led to significantly lower phagocytic rates (by 28.80%, 34.21%, and 11.22%) and total hemocyte counts (by 37.45%, 61.67%, and 46.32%, respectively) (Table 1). Other impacts included increased intracellular reactive oxygen, reduced hemocyte viability, F-actin disruption, and suppressed immune- and cytoskeleton-related gene expression. Higher antibiotic accumulation under co-exposure likely contributed to the enhanced toxicity [45].

Guo et al. [46] used the freshwater bivalve *Corbicula fluminea* to assess the individual and combined toxic effects of fluorescent polystyrene micro- and nanoplastics (6 μm and 80 nm) and the antibiotic ciprofloxacin in sediment. All treatments caused oxidative stress and neurotoxicity, with digestive gland damage linked to impaired free radical scavenging. Filtration rates declined in a dose-dependent manner, likely due to reduced acetylcholinesterase activity. While polystyrene particles lowered ciprofloxacin toxicity in digestive glands by reducing free ciprofloxacin levels, they intensified siphoning inhibition in the nanoplastic co-exposure group (Table 1). Another study examined how two commonly used veterinary antibiotics, oxytetracycline and florfenicol, affected immune function in the blood clam (*Tegillarca granosa*), and whether microplastics influenced these effects [47]. The antibiotics caused significant changes in blood parameters and decreased serum lectin levels. They also increased reactive oxygen species, worsened lipid peroxidation and DNA damage, and reduced hemocyte viability (Table 1). Gene expression analyses showed that immune and detoxification genes were downregulated, while the apoptosis-related gene *Caspase-3* was upregulated. Notably, the presence of microplastics intensified the toxic effects of both antibiotics.

Using *Mytilus coruscus* as a model, Ma et al. [48] explored the combined effects of polystyrene microplastics (80 nm) and norfloxacin on antioxidant and immune-related genes. Microplastics alone or with norfloxacin elevated *CYP3A-1* expression, while *CYP3A-2* was only significantly upregulated at high norfloxacin levels (500 μg/L). *Nrf2* expression also increased in microplastic treatments with no or low norfloxacin. For immune genes, *IRAK-1* was upregulated at low norfloxacin concentrations but suppressed at high concentrations, while the presence of norfloxacin enhanced norfloxacin-induced expression of *IRAK-4* and *HSP70*. Overall, microplastics intensified the impact of norfloxacin on mussel antioxidant and immune responses [48].

### 3.3. Freshwater Fish Models

Zhang et al. [49] explored how polypropylene microplastics influenced the trophic transfer of oxytetracycline from shrimp (*Neocaridina denticulata*) to crucian carp (*Carassius auratus*) using metagenomic analysis. Polypropylene microplastics facilitated the accumulation and transfer of oxytetracycline along the food chain, leading to more severe vacuolation in intestinal cells and eosinophilic necrosis in liver cells. The combination of polypropylene microplastics and oxytetracycline further suppressed intestinal lysozyme activity and complement C3 levels in both species, as well as reduced hepatic immunoglobulin M levels in fish (Table 1). Co-exposure also significantly increased *Actinobacteria* in shrimp and *Firmicutes* in fish, disrupting carbohydrate, amino acid, and energy metabolism [49].

Adding to the understanding of how freshwater species respond to microplastic and antimicrobial exposure, Lu et al. [19] examined the joint effects of polystyrene microplastics and sulfamethoxazole on zebrafish embryos (Figure 3), revealing developmental, physiological, and endocrine disruptions. Co-exposure resulted in increased mortality (25.0%) and malformations (20–35%), along with reduced fetal movement (31.1–37.0%) and swimming activity (26.9–36.8%), and elevated heart rate (19.0–20.9%). Endocrine disruption was evident through raised levels of biomarkers like vitellogenin, 17β-estradiol, testosterone, and triiodothyronine. An antagonistic interaction between polystyrene microplastics and sulfamethoxazole slightly reduced their combined toxicity (Table 1). In another study, two types of aged polystyrene microplastics were produced via UV irradiation and ozonation [50]. At non-teratogenic levels, pristine polystyrene microplastics (80 nm) combined with penicillin significantly reduced zebrafish embryo heartbeats and impaired spontaneous movement, touch response, and swimming behavior. Pristine and UV-aged polystyrene showed similar neurodevelopmental toxicity, while ozonated polystyrene did not. Interestingly, co-exposure with penicillin revealed antagonistic effects for both aged polystyrene types but not for pristine polystyrene. In juvenile fish, exposure to polystyrene microplastics reduced exploratory activity in light/dark tests. Further analysis showed that aged polystyrene with penicillin triggered increased apoptosis, reactive oxygen species production, and neurotransmitter changes (Table 1). UV-aged polystyrene, unlike ozonated polystyrene, also had a higher penicillin adsorption rate, which may explain the greater toxicity observed [50].

Zhang et al. [51] assessed the effects of polystyrene microplastics (10 mg/L) and 3,6-dibromocarbazole (0.5 mg/L), an antimicrobial, on zebrafish embryos. Both alone and in combination, the chemicals increased embryo deformities without affecting mortality or hatching rates. Individually, they elevated reactive oxygen species and triggered apoptosis, but combined exposure reduced these effects, indicating an antagonistic interaction (Table 1). Fluorescent tracing showed that polystyrene microplastics (5 and 50 μm) could not penetrate embryos by 55 h post-fertilization (hpf) due to chorion protection. However, by 96–144 hpf, polystyrene microplastics facilitated the uptake of 3,6-dibromocarbazole and its dioxin-like toxicity in larvae, with 50 μm particles promoting greater accumulation than 5 μm ones [51].

In terms of antifungals, Li et al. [52] conducted a study to examine the individual and combined impacts of polystyrene microplastics (9–70 µm) and the triazole fungicide difenoconazole on zebrafish, aiming to uncover the underlying mechanisms involved. The findings indicated that polystyrene microplastics partially decreased the accumulation of difenoconazole in zebrafish and mitigated the oxidative stress it caused in the liver (Table 1). Analysis of transcriptome data and quantitative reverse transcription polymerase chain reaction results identified several biological pathways linked to the zebrafish response to difenoconazole, with polystyrene microplastics appearing to moderate the associated gene expression alterations. Additionally, Bhagat et al. [53] examined how polystyrene nanoparticles (50 nm) influenced the toxicity of the antifungal agents ketoconazole and fluconazole in zebrafish embryos. When embryos were co-exposed to polystyrene nanoplastics (1 mg/L) and ketoconazole or fluconazole (1 mg/L) for 96 h, a decline in hatching, survival, and heart rates was observed, accompanied by an increase in malformations and catalase activity (Table 1). The bax/bcl2 ratio, a key indicator of apoptosis, was elevated after exposure to either polystyrene nanoplastics, ketoconazole, or fluconazole alone, but was significantly higher when combined exposure occurred [54]. These findings suggest that polystyrene nanoplastics intensified the toxicity of azole antifungals by promoting reactive oxygen species production, enhancing lipid peroxidation, and disrupting the expression of genes involved in oxidative stress and cell death.

Wu et al. [55] assessed developmental and toxicological effects of polyethylene microplastics and tetracycline on zebrafish (*Danio rerio*) embryos and larvae by analyzing various biomarker responses. Co-exposure to tetracycline and polyethylene microplastics caused significant physiological disruptions, including reduced spontaneous heartbeats, heart toxicity, increased mortality in a dose-dependent manner, shortened body length, and noticeable physical deformities (Table 1). Mechanistic investigations showed that the buildup of reactive oxygen species altered enzyme activities, leading to abnormal blood vessel formation, strong inflammatory reactions, and disrupted gene expression. Xiong et al. [56] examined how co-exposure to 20 μm or 80 nm microplastics and sulfamethazine affected zebrafish at larval and adult stages. In larvae, liver damage intensified, with fewer hepatocytes, macrophages, and neutrophils observed (Table 1). Meanwhile, the expression of inflammatory cytokines and antioxidant enzyme activity increased. In adults, transcriptome data revealed that co-exposure altered genes associated with oxidative stress, inflammation, and mitogen-activated protein kinase (MAPK) signaling. The combined exposure also triggered liver cell apoptosis and reduced cell proliferation, which was linked to Nrf2 translocation into the nucleus and elevated Nrf2 and NF-κB p65 protein levels.

Similarly, polystyrene microplastics were observed to increase the accumulation of roxithromycin in red tilapia tissues compared to exposure to roxithromycin alone [21]. When fish were exposed to 100 μg/L polystyrene microplastics and 50 μg/L roxithromycin, the highest roxithromycin concentrations detected were 39,672.9 μg/kg in the gut, 1767.9 μg/kg in the gills, 2907.5 μg/kg in the brain, and 4307.1 μg/kg in the liver. Interestingly, after 14 days, the neurotoxic effects of roxithromycin were reduced in the presence of polystyrene microplastics. Liver enzyme activities linked to cytochrome P450, specifically ethoxyresorufin-O-deethylase and benzyloxy-4-(trifluoromethyl)coumarin-O-debenzyloxylase, varied notably in co-exposure groups compared to roxithromycin alone, implying that microplastics may alter roxithromycin metabolism in tilapia. Additionally, co-exposure led to a marked increase in superoxide dismutase activity and a decrease in malondialdehyde levels, indicating that polystyrene microplastics helped reduce oxidative stress in the liver during combined exposure (Table 1) [21].

Zhang et al. [57] further demonstrated that aged microplastics enhanced roxithromycin accumulation in the tissues of *Carassius auratus* (Figure 3) in a size-dependent manner. Both aged microplastics and roxithromycin significantly increased liver and gut antioxidant enzyme activities (superoxide dismutase and catalase) and suppressed brain acetylcholinesterase activity (Table 1). Smaller aged microplastics intensified roxithromycin-induced damage in the liver, gills, and brain, while larger aged microplastics caused more severe intestinal injury. High-throughput 16S rRNA sequencing showed increased abundance of Proteobacteria in the 0.5 μm aged microplastics + roxithromycin group, and higher levels of Firmicutes and Bacteroidota in the 50 μm aged microplastics + roxithromycin group. Metabolomic analysis revealed that aged microplastics and roxithromycin exerted size-dependent, long-term impacts on gut microbial metabolites, particularly affecting galactose metabolism, amino acid metabolism, and primary bile acid biosynthesis after a 7-day depuration period [57].

Additionally, a study examined how oxytetracycline and polystyrene microplastics affected growth, intestinal health, and gut microbiota in juvenile yellow catfish (*Pelteobagrus fulvidraco*) (Figure 3) over 28 days [58]. Fish were exposed to oxytetracycline (500 ng/L), low (100 μg/L) and high (1000 μg/L) concentrations of polystyrene microplastics or their combinations. Neither oxytetracycline nor 100 μg/L microplastics alone significantly impacted growth, antioxidant and digestive enzyme activities, or gut microbial diversity. However, combined exposure to oxytetracycline and 100 μg/L microplastics elevated antioxidant enzymes (superoxide dismutase, catalase), caused mild intestinal damage, and increased Proteobacteria abundance (Table 1). Catalase activity was also higher than in the oxytetracycline-only group. In contrast, 500 μg/L microplastics alone suppressed growth, impaired digestion, triggered oxidative stress, and reduced Firmicutes levels. These adverse effects were more severe under combined oxytetracycline and 500 μg/L microplastics exposure, indicating synergistic toxicity [58].

### 3.4. Marine Fish Models

In addition to freshwater studies, marine species such as *Pomatoschistus microps* have also been evaluated for combined microplastic and antibiotic toxicity. Fonte et al. [59] found that combined exposure to cefalexin and microplastics reduced post-exposure predatory performance in *P. microps*, especially at cefalexin concentrations ≥1.25 mg/L at 20 °C. At 25 °C, the toxicity of cefalexin alone and with microplastics significantly differed, with 96 h-EC_50_ values of 3.8 and 5.2 mg/L, respectively, indicating interaction effects on all biomarkers. Rising temperature from 20 °C to 25 °C intensified microplastic-induced mortality (from 8% to 33%) and cefalexin’s inhibitory effects on post-exposure predatory performance (up to 70%). At 25 °C, the mixture also elevated acetylcholinesterase activity by 14% and lipid peroxidation by 72% (Table 1). Overall, temperature increased the toxic effects of both pollutants, individually and combined [59].

Zhang et al. [60] demonstrated that simultaneous exposure to aged polylactic acid microplastics and the antibiotic sulfamethazine resulted in significant weight gain (ranging from 20.9% to 26.2%) and symptoms resembling fatty liver disease in juvenile marine medaka (*Oryzias melastigma*) (Figure 3). The findings suggest that sulfamethazine serves as a trigger or initiator, while polylactic acid exerts selective environmental pressure that influences the composition of gut microbial communities. These microbial changes enhance the degradation of polylactic acid into lactic acid, which in turn stimulates gluconeogenesis in the intestine, increasing glucose production. The resulting glucose surplus disrupts the balance between glucose and fat metabolism in the liver, promoting fat storage in the form of triglycerides and lipid droplets, ultimately contributing to obesity in the fish (Table 1) [60]. In another study, *O. melastigma* were exposed for four weeks to polystyrene microplastics (10 μm, 0.1% *w*/*w* in food) and tetracyclines (50 μg/L in water), both individually and in combination [61]. The presence of microplastics did not significantly affect the availability of tetracyclines in water. However, fish exposed to tetracyclines alone or in combination with microplastics showed reduced weight gain and lower liver lipid content compared to controls (Table 1). Body length growth was further suppressed in the co-exposure group relative to single exposures. Both treatments altered gut microbiota structure and diversity, with tetracycline and the combined exposure showing similar microbiota profiles that differed notably from those of the polystyrene microplastics and control groups (Figure 3) [61].

### 3.5. Other Aquatic Animals

Wu et al. [62] investigated the effects of exposure to polystyrene microplastics and tetracycline, both individually and in combination, on cellular apoptosis, oxidative stress, and metabolic functions in *Aurelia aurita* polyps (Figure 3). The study showed that apoptosis levels were significantly elevated in the group exposed to both microplastics and tetracycline compared to the control and single-exposure groups (Table 1). Tests assessing antioxidant capacity revealed that oxidative damage persisted in the microplastics-only group even after a 288 h recovery period. Metabolomic analysis uncovered distinct physiological responses to microplastics and tetracycline, including alterations in key biological pathways such as ATP-binding cassette transporters and protein digestion, as well as disturbances in various metabolites like antioxidants and neurotransmitters. Interestingly, tetracycline displayed a non-linear toxicity pattern, with higher concentrations not always causing greater damage. Moreover, in combined exposure scenarios, tetracycline unexpectedly reduced oxidative stress, likely due to its antibacterial activity [62].

Huang et al. [63] examined the toxic effects of norfloxacin (0–5 μg/L) and polystyrene nanoparticles (10^4^ particles/L) on *Tachypleus tridentatus* by assessing oxidative stress, molting genes, and gut microbiota. Short-term (7-day) exposure to either or both pollutants disrupted redox balance, as shown by increased malondialdehyde levels. Over longer exposure (14–21 days), malondialdehyde normalized as catalase and glutathione levels rose, though superoxide dismutase remained unchanged. Gene expression related to molting and stress responses showed limited changes. Both pollutants also altered the gut microbiota, notably increasing the abundance of Bacteroidetes. A summary of the combined toxic effects on aquatic animals is provided in Table 1 below.

**Table 1:** Combined toxicity of microplastics and antimicrobials on aquatic animals.

Organism Type	Species	Microplastics + Antimicrobials	Main Effects	Reference
Crustaceans	*Daphnia magna*	Polystyrene microplastics (1 and 10 μm) + roxithromycin (0.01 mg/L)	Co-exposure reduced glutathione peroxidase activity, lowered malondialdehyde levels (1 μm), decreased glutathione S-transferase activity (10 μm); oxidative stress modulation; 1 μm microplastics mitigated roxithromycin toxicity	[22]
	*Daphnia magna*	UV-aged polystyrene microplastics (0.1, 10 µg/L) + roxithromycin	Increased F0 survival (20–40%), mitigated reproductive toxicity in F0; in F1, reproductive toxicity worsened; co-exposure shifted swimming inhibition to stimulation; elevated acetylcholinesterase activity (1.61–3.25×), increased oxidative damage	[41]
	*Daphnia magna*	5.8 µm polystyrene microplastics (≥2 mg/L) + triclosan, triclocarban, or methyl-triclosan	Chronic exposure delayed first brood, reduced brood frequency and offspring; polystyrene enhanced antimicrobial reproductive toxicity; methyl-triclosan + polystyrene caused greatest population decline	[42]
	*Ceriodaphnia dubia*	1 µm polystyrene microplastics + acyclovir or imidacloprid	Short-term antagonistic genotoxicity; reproduction decreased by 54.8% at 0.15 µg/L polystyrene + 0.0379 µg/L acyclovir; chronic toxicity increased at low concentration	[43]
	*Procambarus clarkii*	Polystyrene microplastics (100 mg/L) + pyrogallol (10 mg/L)	Altered hemocyte counts; liver enzymes increased; antioxidant enzymes decreased; histological damage	[44]
Mollusks	*Mytilus coruscus*	Polystyrene microplastics + norfloxacin (≤500 µg/L)	Upregulated antioxidant genes (CYP3A-1, Nrf2); immune genes altered (IRAK-1, IRAK-4, HSP70); microplastics intensified norfloxacin effects on antioxidant and immune responses	[48]
	Thick-shelled mussels	Polystyrene microplastics (0.26 mg/L, 500 nm) + oxytetracycline (270 ng/L), florfenicol (42 ng/L), or sulfamethoxazole (140 ng/L)	Co-exposure impaired immune function: reduced phagocytic rates (11–34%), total hemocyte counts (37–62%), increased reactive oxygen species, disrupted cytoskeleton, suppressed immune/detoxification gene expression	[45]
	*Corbicula fluminea*	Polystyrene microplastics/nanoplastics + ciprofloxacin	Oxidative stress, neurotoxicity, digestive gland damage; reduced filtration rate; polystyrene lowered ciprofloxacin toxicity in digestive gland but increased siphoning inhibition in nano-polystyrene + antibiotic group	[46]
	*Tegillarca granosa*	Polystyrene microplastics + oxytetracycline, or florfenicol	Reactive oxygen species increased; immune gene suppression; DNA damage; hemocyte viability decreased; apoptosis gene upregulated	[47]
Freshwater fish	*Carassius auratus*	Polystyrene microplastics + oxytetracycline	Liver and intestinal damage; immune suppression	[49]
	*Danio rerio*	Polystyrene microplastics + sulfamethoxazole	Increased mortality (25%), malformation (20–35%), reduced fetal movement (31–37%), swimming activity (27–37%), elevated heart rate (19–21%); endocrine disruption (vitellogenin, 17β-estradiol, testosterone, T3); slight antagonistic effect	[19]
	*Danio rerio*	Aged polystyrene microplastics + penicillin	Pristine and UV-aged polystyrene + penicillin reduced heartbeat, impaired movement; ozonated polystyrene had no effect; co-exposure showed antagonistic effects with aged polystyrene	[50]
	*Danio rerio*	Polystyrene microplastics (10 mg/L) + 3,6-dibromocarbazole (0.5 mg/L)	Embryo deformities increased; reactive oxygen species decreased in co-exposure	[51]
	*Danio rerio*	Polystyrene microplastics + difenoconazole	Reduced difenoconazole accumulation; mitigated liver oxidative stress; moderated gene expression changes	[52]
	*Danio rerio*	Polystyrene microplastics (1 mg/L) + ketoconazole or fluconazole (1 mg/L)	Reduced hatching, survival, and heart rates; increased malformations, catalase activity, bax/bcl2 ratio; polystyrene intensified azole antifungal toxicity via reactive oxygen species and apoptosis	[53]
	*Danio rerio*	Polyethylene microplastics + tetracycline	Reduced heartbeats, heart toxicity; increased mortality; shortened body length, deformities; elevated reactive oxygen species; inflammatory response; altered gene expression	[55]
	*Danio rerio*	Microplastics + sulfamethazine	Larvae: liver damage, macrophage/neutrophil reduction, elevated inflammatory cytokines and antioxidant activity; adults: altered oxidative stress, inflammation, MAPK signaling, liver apoptosis	[56]
	*Oreochromis* sp.	Polystyrene microplastics (100 µg/L) + roxithromycin (50 µg/L)	Increased tissue roxithromycin accumulation; reduced neurotoxicity; altered CYP450 liver enzyme activities; increased superoxide dismutase, decreased malondialdehyde; oxidative stress mitigation	[21]
	*Carassius auratus*	Aged microplastics + roxithromycin	Increased liver/gut antioxidant activity; suppressed brain acetylcholinesterase; smaller microplastics caused more liver/gill/brain damage; larger microplastics caused more intestinal injury; altered gut microbiota/metabolites	[57]
	*Pelteobagrus fulvidraco*	Polystyrene microplastics (100 or 500 µg/L) + oxytetracycline (500 ng/L)	100 μg/L + oxytetracycline: mild intestinal damage, increased superoxide dismutase and catalase, higher Proteobacteria; 500 μg/L + oxytetracycline: suppressed growth, digestion impairment, oxidative stress, reduced Firmicutes; synergistic toxicity	[58]
Marine fish	*Pomatoschistus microps*	Polystyrene microplastics + cefalexin (≥1.25 mg/L)	Predation decreased; acetylcholine and lipid peroxidation increased; temperature-dependent	[59]
	*Oryzias melastigma*	Polylactic acid microplastics + sulfamethazine	Weight gain (20.9–26.2%); fatty liver symptoms; gut microbiota altered	[60]
	*Oryzias melastigma*	Polystyrene microplastics (0.2% *w*/*w* in food) + tetracyclines (50 µg/L)	Weight gain and liver lipid decreased; suppressed body length growth; gut microbiota altered	[61]
Other aquatic taxa	*Aurelia aurita*	Polystyrene microplastics + tetracycline	Apoptosis increased; oxidative stress; metabolic disruption	[62]
	*Tachypleus tridentatus*	Polystyrene nanoplastics (10^4^ particles/L) + norfloxacin (0–5 µg/L)	Oxidative stress; microbiota Bacteroidetes increased	[63]

### 3.6. Implications

The interactions between microplastics and antimicrobials in aquatic environments present a complex toxicological landscape, with implications varying widely across species, particle characteristics, and exposure contexts. Microplastics, particularly those that are aged or of smaller particle size, often act as carriers for antimicrobial compounds, altering their environmental fate and bioavailability [64]. Microplastics alter the bioavailability of antibiotics by adsorbing them onto their surfaces, which can reduce their free concentrations in water but prolong their persistence. At the same time, the plastisphere provides microenvironments that concentrate antibiotics and microbes, potentially enhancing local exposure and selective pressure for resistance [65]. Liu et al. [66] revealed that after incubating for 60 days, microplastics hosted more diverse microbial communities than the surrounding water. Photoaging reduced biofilm attachment, while biofilms promoted sulfamethazine release and bioavailability. Aged microplastics, due to their increased surface area and oxygen-containing functional groups, enhance the sorption and subsequent transport of antimicrobials, leading to greater tissue accumulation in aquatic organisms [67]. For instance, aged polystyrene microplastics have been shown to increase roxithromycin accumulation in fish and invertebrate tissues, demonstrating their role in modifying toxicokinetics and potentially facilitating broader ecological exposure [41,57].

Co-exposure to microplastics and antimicrobials frequently results in additive or synergistic toxicity, amplifying adverse effects on physiological, reproductive, and developmental processes. Across a range of taxa, including crustaceans, mollusks, and fish, combined exposure has been consistently linked to elevated oxidative stress, increased lipid peroxidation, disruption of enzymatic antioxidant defenses, and induction of apoptosis. For example, *Daphnia magna* exposed to microplastics and triclosan or methyl-triclosan exhibited more severe reproductive toxicity than under single exposures, and zebrafish embryos co-exposed to microplastics and antifungal agents showed increased malformations, suppressed heart rates, and altered gene expression profiles associated with oxidative damage and cell death [19,42,50,55]. Similarly, mollusks displayed heightened immunotoxicity when exposed to both microplastics and antibiotics, marked by reduced phagocytic capacity, hemocyte viability, and immune-related gene expression [45,47].

However, certain combinations can exhibit antagonistic effects, with microplastics mitigating rather than intensifying toxicity under specific conditions, although this is less frequently reported. This antagonism often arises from microplastics adsorbing antimicrobials and thereby reducing their free concentration or chemical reactivity. For instance, sulfamethoxazole and microplastics displayed reduced toxicity when co-administered to zebrafish embryos, and acyclovir combined with microplastics produced weaker genotoxic responses in short-term tests on crustaceans [19,43]. Yet such interactions are nuanced and highly dependent on the physicochemical properties of the pollutants, their concentrations, and the biological context.

Oxidative stress and inflammation emerge as central pathways in the combined toxicity of microplastics and antimicrobials. Most studies report elevated levels of oxidative damage biomarkers, such as malondialdehyde, and disrupted activities of antioxidant enzymes like catalase and superoxide dismutase. Co-exposure also frequently activates inflammatory signaling and apoptosis, as reflected in the upregulation of genes involved in stress responses (e.g., *Nrf2*, *NF-κB*, *MAPK*) and downregulation of detoxification and immune functions [22,56,68]. These molecular disruptions underpin broader physiological impairments, including reduced reproduction, growth inhibition, and immune suppression.

### 3.7. Limitations and Future Directions

Despite growing evidence of the interactive toxicity between microplastics and antimicrobials in aquatic systems, several limitations constrain the generalizability and mechanistic understanding of current findings. Most studies use laboratory-based exposures under static or semi-static conditions, with concentrations of microplastics and antimicrobials often exceeding environmentally relevant levels. Such overdosing can skew results in several ways: microplastics at unrealistically high levels can physically stress organisms or dilute their food supply, while high microplastic loads can also bind large amounts of antimicrobials, altering the actual dissolved concentrations and producing misleading toxicity patterns. These artifacts may lead to overestimation of risks or to apparent interactions (synergistic or antagonistic) that are not representative of natural systems [69]. To improve ecological relevance, future research should adopt tiered exposure ranges that reflect ambient, hotspot, and worst-case conditions, use environmentally aged rather than pristine microplastics, and report both nominal and measured concentrations of antimicrobials. Setting realistic thresholds for microplastic particle numbers, surface area, and antimicrobial concentrations, while ensuring that sorption or physical artifacts do not dominate, would help generate more reliable insights into the true interactive toxicity of these pollutants. For instance, a water-only factorial design is a straightforward way to examine how microplastics and antimicrobials interact. Organisms are exposed to a matrix of treatments combining different levels of each stressor, for example, microplastics at 10, 1000, and 100,000 particles per liter crossed with antimicrobials at 10, 100, and 1000 ng/L. This setup makes it possible to detect not only individual effects but also whether the combined effects are additive, synergistic, or antagonistic. Importantly, keeping exposures within these tiers ensures ecological relevance.

Research has disproportionately focused on a few model species (e.g., *Daphnia magna*, zebrafish, and bivalves) and early developmental stages. Research is limited on how microplastics move through the food chain, such as from aquatic invertebrates to fish. This trophic transfer introduces microplastics-bound antimicrobials into higher organisms, where they may accumulate and disrupt gut microbiota, induce toxicity, or promote antibiotic resistance [49]. Such transfers highlight the potential for microplastics to act as long-term vectors of antimicrobials across food webs, posing ecological risks and potential threats to wildlife and human health. Responses in other trophic levels (e.g., predators, decomposers), benthic species, and adult or reproductive phases remain understudied, limiting the ecological relevance of toxicity assessments.

Microplastics used in experiments are often pristine, spherical, and uniform in size, which does not reflect the heterogeneity of environmental microplastics in terms of shape, size, polymer type, aging status, and presence of additives. Polystyrene microplastics are more thoroughly studied than other microplastic types. Focusing mainly on polystyrene microplastics limits the applicability of findings, since different polymers vary in density, crystallinity, surface charge, and hydrophobicity. These properties strongly influence how microplastics behave in water (e.g., floating vs. sinking, aggregation, aging) and how readily they sorb or release antimicrobials. For instance, polyethylene and polypropylene are more buoyant and hydrophobic, while polyamide, polyvinyl chloride, and polyethylene terephthalate may carry more surface charge or additives, altering bioavailability and interaction with organisms [70]. As a result, toxicity patterns observed for polystyrene cannot be assumed to hold for all polymers, and extrapolation should be made cautiously. Expanding studies to include a wider range of polymer types would provide a more realistic picture of how microplastics contribute to combined toxicity in aquatic systems.

Short-term exposures dominate the literature, while long-term ecological effects, multigenerational responses, and population-level impacts are rarely addressed. This gap hinders the ability to evaluate potential evolutionary adaptations, reproductive trade-offs, or ecosystem-level consequences of prolonged co-exposure. Furthermore, natural environments often contain mixtures of multiple pollutants. Yet most studies examine binary combinations (one microplastic type and one antimicrobial), ignoring the interactions with other contaminants such as heavy metals, pesticides, hormones, or nutrients. These mixtures could produce antagonistic, synergistic, or cumulative effects not captured in simplified studies.

To enhance understanding and improve risk assessment of combined microplastic-antimicrobial toxicity in aquatic systems, future studies should use environmentally relevant concentrations and incorporate aged, weathered, and irregularly shaped microplastics that better reflect field conditions. Fluctuating environmental parameters (e.g., temperature, pH) should be included to mimic real-world exposure. Investigations should encompass a broader range of aquatic animals at different trophic levels. Beyond biochemical and developmental endpoints, studies should assess behavioral, reproductive, immunological, and ecological fitness indicators under chronic and multigenerational conditions.

To reflect realistic pollution scenarios, studies should include combinations of microplastics with multiple antimicrobials and other co-occurring contaminants (e.g., metals, pesticides, other emerging pollutants). Mixture toxicity models and interaction-based frameworks (e.g., concentration addition, independent action) should be applied to interpret combined effects. Research is needed to trace the fate and effects of microplastics-associated antimicrobials through food webs. Studies should quantify bioaccumulation, biomagnification, depuration rates, and transformation products across trophic levels, particularly in species of commercial and ecological significance.

To improve comparability across studies, standardized protocols for microplastic aging and characterization should include (i) using well-defined polymers, sizes, and shapes with thorough baseline characterization (e.g., Fourier Transform Infrared/Raman, size distribution, surface charge, hydrophobicity, crystallinity); (ii) applying reproducible aging methods such as controlled UV/photooxidation, thermal, chemical, mechanical, or biofouling treatments with clearly reported conditions; (iii) re-characterizing microplastics after aging to assess surface chemistry, morphology, biofilm formation, and leachates; and (iv) using standardized exposure media, dispersion methods, and contamination controls. This framework would harmonize experimental designs and enable reliable cross-study comparisons.

## 4. Toxicity on Terrestrial Animals

The combined toxicity of microplastics and antimicrobials on terrestrial organisms is an emerging area of environmental concern. While much attention has focused on aquatic systems, recent evidence indicates that soil environments are also increasingly contaminated with these co-occurring pollutants due to agricultural runoff, sewage sludge application, and plastic waste degradation [71,72,73]. In terrestrial ecosystems, microplastics can interact with antimicrobials by adsorbing or transporting them, potentially altering their bioavailability and persistence [4,74]. These interactions may intensify toxic effects on soil-dwelling organisms such as earthworms and insects, leading to disruptions in growth and reproduction [75]. For simplicity, amphibians and waterfowl are discussed in this section for illustration purposes.

### 4.1. Rodent Models

**Figure 4:**
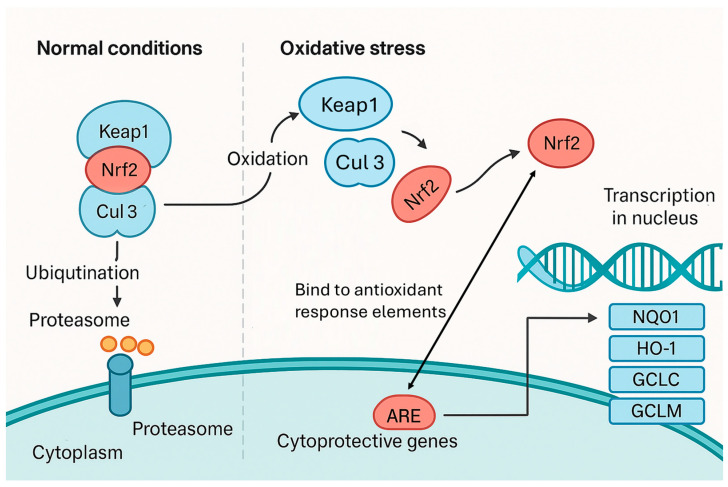
Keap1-Nrf2 signaling pathway. Under normal conditions, Keap1-Nrf2-Cul3 complex facilitates ubiquitination of Nrf2, and ubiquitinated Nrf2 is degraded by the proteasome in the cytoplasm, preventing excessive antioxidant gene activation. During oxidative stress, Nrf2 is released from the complex, translocates into the nucleus, binds with ARE, and activates the transcription of cytoprotective genes (NQO1, HO-1, GCLC, and GCLM).

Using a mouse model to simulate human physiology, Fu et al. [76] found that simultaneous exposure to sulfamethoxazole, a commonly used antibiotic, and polystyrene microplastics led to significant accumulation in detoxifying organs. Specifically, the liver accumulated up to 41.70 μg/kg of sulfamethoxazole, while 3.83% of the ingested microplastics were retained in the kidneys. Histological analysis of the liver showed tissue damage such as amyloidosis and necrocytosis. Compared to exposure to sulfamethoxazole alone, co-exposure with microplastic-bound sulfamethoxazole markedly increased malonaldehyde and NF-κβ levels by 174% and 104%, respectively, while the activity of key antioxidant enzymes declined by as much as 22% (Table 2). These results indicate that sulfamethoxazole adsorbed onto reactive microplastic surfaces intensifies liver toxicity. Gene expression analysis further revealed that oxidative and inflammatory damages were linked to the overexpression of *Nrf2*, leading to disruption of the Keap1–Nrf2 signaling pathway (Figure 4) [76]. Increasing Nrf2 activity has been reported to boost the survival and growth of cancer cells and to impact immune homeostasis [77,78].

Expanding the understanding of co-exposure risks in early life stages, Xia et al. [79] established a juvenile mouse model to mimic dietary exposure to polystyrene microplastics and tetracycline over an 8-week period. The study found that simultaneous exposure led to the most pronounced impairment of the intestinal barrier, primarily due to heightened inflammation and oxidative stress. This co-exposure also diminished populations of beneficial probiotic bacteria while encouraging the proliferation of opportunistic pathogens (Table 2). Metagenomic analysis revealed an increased presence of microbial species harboring ARGs and virulence factor genes under combined exposure conditions, suggesting a higher risk of spreading harmful genetic elements [79].

Further evidence of synergistic effects comes from a study in which mice were orally exposed to polystyrene microplastics (5 µm, at doses of 0.012 or 0.120 mg/kg) and/or epoxiconazole (0.080 mg/kg) over six weeks [80]. The results revealed that co-exposure to the higher dose of polystyrene (0.120 mg/kg) and epoxiconazole led to more pronounced tissue damage, functional impairment, oxidative imbalance, and metabolic disruptions than exposure to either pollutant alone. Notably, this enhanced toxicity correlated with greater tissue accumulation of both substances. Further analysis showed that epoxiconazole disrupted the gut barrier by altering the gut microbiota, which facilitated increased absorption and accumulation of polystyrene (Table 2). This, in turn, impaired the liver’s ability to metabolize and eliminate epoxiconazole [80]. Overall, the study demonstrated that polystyrene microplastics and epoxiconazole exerted synergistic toxic effects in mice, driven by mutual enhancement of bioaccumulation and metabolic interference.

Sun et al. [81] found that doxycycline, combined with exposure to polystyrene microplastics, perturbed gut microbiota homeostasis in mice, which mediated brain lesions and inflammation, resulting in a concomitant decline in learning and memory behaviors through the gut–brain axis. Of note, exposure to polystyrene microplastics resulted in intestinal damage and structural change, but doxycycline did not accelerate the disruption of intestinal barrier integrity in microplastics-treated mice (Table 2). This implies that microplastics, as well as their associated plastisphere and antimicrobials, may influence microbial balance and chemical–microbe interactions in the digestive systems of organisms. Interestingly, fecal microbiota transplantation can reverse neurological impairment caused by combined polystyrene microplastics and doxycycline exposure via compensating gut microbes; therefore, the learning and memory abilities of mice were also recovered [81].

### 4.2. Amphibians

Beyond mammalian models, amphibians have also been used to assess combined pollutant effects. Zhang et al. [82] reported that polystyrene microplastics intensified the adverse effects of levofloxacin on the growth and development of *Rana nigromaculata*. The degree of developmental inhibition followed this order: levofloxacin–microplastics (10 µm) > levofloxacin–microplastics (0.1 µm) > levofloxacin–microplastics (1 µm). Microplastics sized 0.1 and 1 μm were capable of crossing the blood–brain barrier, where they interacted with levofloxacin and disrupted growth and development by altering thyroid axis function (Table 2). Moreover, co-exposure to levofloxacin and microplastics resulted in greater disruption of the thyroid axis than exposure to levofloxacin alone [82].

In another connected study by Zhang et al. [83], a 45-day exposure to levofloxacin and microplastics of various sizes revealed that levofloxacin caused neurotoxicity in *Rana nigromaculata* tadpoles, with the most severe behavioral effects observed in levofloxacin–microplastics (10 μm), followed by microplastics of 0.1 μm and microplastics of 1 μm. Transcriptomic analysis showed that co-exposure with 0.1 and 10 μm microplastics disrupted neural function via the cell adhesion molecule signaling pathway (Table 2). However, intestinal oxidative stress responses did not follow the same pattern as behavioral outcomes. The gut bacterium *Parabacteroides* emerged as a potential bioindicator of microplastic exposure. Targeted intestinal neurotransmitter analysis suggested that in the levofloxacin–microplastics (1 μm) group, levofloxacin mitigated the dysbiosis caused by 1 μm microplastics by modulating gut microbes involved in the tricarboxylic acid cycle, gluconeogenesis, and tetrapyrrole biosynthesis. This led to altered levels of key metabolites (e.g., reduced methionine and ornithine, increased 5-hydroxyindoleacetic acid), which may have helped alleviate neurotoxicity [83].

### 4.3. Avians

A study exposed Muscovy ducks to polystyrene microplastics, chlortetracycline, and their combination for 56 days to assess intestinal impacts [84]. Microplastics reduced chlortetracycline accumulation in the liver and intestines but increased its excretion in feces. Microplastics alone triggered oxidative stress, inflammation, and intestinal barrier damage, along with gut microbiota imbalance, marked by increased *Streptococcus* and *Helicobacter*. Interestingly, combined exposure to microplastics and chlortetracycline mitigated some intestinal damage by modulating the gut microbiome (Table 2) [84].

### 4.4. Earthworm Models

Terrestrial invertebrates, such as earthworms, offer important insights into soil ecotoxicology. A co-exposure study showed that earthworms exposed to polyethylene microplastics sized 13 µm (0.25% *w*/*w*) accumulated the greatest amount of tebuconazole on day 7–19.77% more than those exposed to tebuconazole alone, 7.27% more than the 0.25% (*w*/*w*) 48 µm microplastics group, and 10.30% more than the 0.25% (*w*/*w*) 150 µm microplastics group [85]. Among the treatments, the 13-µm microplastics induced the strongest oxidative stress, as evidenced by significant elevations in the oxidative stress markers catalase and peroxidase. By day 28, earthworms in the 13 µm microplastics group exhibited the lowest levels of glutathione sulfotransferase gene expression, while genes associated with antibacterial defense, i.e., heat shock protein 70 and translationally controlled tumor protein, were most highly expressed [85]. These results suggest that exposure to 13-µm microplastics caused considerable DNA damage and heightened toxicity in the earthworms (Table 2).

In another earthworm study, Yang et al. [86] demonstrated that polyethylene nanoplastics extended the environmental persistence of pyraclostrobin in soil by 13 days, led to an 8.4% increase in pyraclostrobin accumulation within *Eisenia fetida*, and caused a 26.8% reduction in their body weight. While exposure to pyraclostrobin alone or in combination with polyethylene nanoplastics significantly elevated the diversity of the gut microbiota in earthworms, polyethylene nanoplastics alone reduced microbial diversity but increased the relative proportions of Proteobacteria and Firmicutes. Additionally, both pyraclostrobin and polyethylene nanoplastics, individually and together, promoted a higher diversity and abundance of ARGs within earthworm guts, expanded the range of microbial hosts carrying ARGs, and intensified the complexity of the networks involving ARGs and antibiotic-resistant bacteria (Table 2). The number of contigs containing plasmid-derived ARGs was 1.5 times higher in the pyraclostrobin group, 3.8 times higher in the polyethylene nanoplastic group, and 2.4 times higher in the combined treatment, compared to the control [86].

Bao et al. [87] explored how microplastics of different aging stages act as carriers for the fungicide azoxystrobin in water, epidermal mucus of earthworms, and simulated intestinal fluid, as well as the associated toxicity. Findings showed that aged microplastics had increased surface area and more oxygen-containing functional groups. All types of microplastics could quickly adsorb and release azoxystrobin across the three environments, with UV-aged microplastics showing a greater adsorption capacity in water and mucus. Although aged microplastics exhibited significantly higher desorption capacity than unaged ones, their desorption rates remained similar. Toxicity tests revealed that UV-aged microplastics with azoxystrobin had the lowest 96 h (median lethal concentration) LC_50_, indicating higher toxicity. These fungicide-loaded microplastics triggered oxidative stress, caused damage to the earthworms’ skin and intestinal tissues, and impaired digestion and barrier functions (Table 2). Moreover, the desorption of azoxystrobin in mucus and intestinal fluid was strongly associated with the observed toxic effects in earthworms [87].

Complementing the above findings, Sun et al. [88] revealed that the soil-bioaccumulation factor of dufulin in earthworms peaked on day 14, and the presence of microplastics notably enhanced the accumulation of dufulin within the organisms. Biochemical assessments indicated that exposure to dufulin alone led to observable oxidative stress in earthworms by day 14, whereas co-exposure to dufulin and microplastics triggered oxidative damage as early as day 7, which persisted through day 14. Metabolomic profiling demonstrated that dufulin exposure alone significantly impacted the levels of 14 metabolites and disrupted two metabolic pathways. However, when earthworms were exposed to both dufulin and microplastics, changes were observed in 21 metabolites and 3 metabolic pathways (Table 2) [88]. These findings suggest that microplastics intensify both the oxidative stress and metabolic disturbances induced by dufulin in earthworms.

### 4.5. Enchytraeids

Broadening the scope to another terrestrial invertebrate, Ma et al. [89] demonstrated that tetracycline accumulated significantly in *Enchytraeus crypticus* when the organisms were exposed to tetracycline alone or together with microplastics, compared to the control or microplastics-only groups. However, no significant differences in tetracycline accumulation were observed among the tetracycline, polyamide + tetracycline, and polyvinyl chloride + tetracycline treatments. Both tetracycline and microplastics caused notable disruptions to the microbial community and significantly reduced the alpha diversity of *E. crypticus* microbiota. Despite these effects, there were no significant differences in microbial diversity between the individual and combined treatments, and no evidence of synergistic toxicity from the combined exposure in terms of microbiome diversity. All exposure groups, however, showed an increase in the diversity of ARGs, ranging from 39 to 49 types, compared to 25 types detected in the control group [89]. A summary of the combined toxic effects on terrestrial animals is presented in Table 2 below.

**Table 2:** Combined toxicity of microplastics and antimicrobials on terrestrial animals.

Organism Type	Species	Microplastics + Antimicrobials	Main Effects	Reference
Rodent	Mouse	Polystyrene microplastics + sulfamethoxazole	Increased sulfamethoxazole accumulation in liver (41.7 μg/kg); microplastics retained in kidneys (3.83%); liver tissue damage (amyloidosis, necrocytosis); increased malonaldehyde (174%) and NF-κβ (104%); decreased antioxidant enzymes (22%); disrupted Keap1–Nrf2 signaling; enhanced oxidative stress and inflammation	[76]
	Juvenile mouse	Polystyrene microplastics + tetracycline	Impaired intestinal barrier; increased inflammation and oxidative stress; decreased probiotics, increased opportunistic pathogens; increased ARGs and virulence genes in microbiota	[79]
	Mouse	Polystyrene microplastics + epoxiconazole	Co-exposure (0.120 mg/kg microplastics) caused synergistic toxicity: increased tissue damage, oxidative imbalance, metabolic disruption; mutual enhancement of bioaccumulation; gut barrier disruption, causing increased absorption of microplastics and antifungal pesticide	[80]
	Mouse	Polystyrene microplastics + doxycycline	Gut microbiota disruption causing brain lesions, inflammation, decreased learning and memory; intestinal barrier damage by microplastics (not accelerated by doxycycline); fecal microbiota transplant restored cognitive functions	[81]
Amphibian	*Rana nigromaculata*	Polystyrene microplastics (0.1–10 µm) + levofloxacin	Growth and development inhibition (size-dependent); microplastics crossed blood–brain barrier; thyroid axis disruption stronger with co-exposure; neurotoxicity	[82]
	*Rana nigromaculata* (tadpoles)	Polystyrene microplastics (0.1, 1, 10 µm) + levofloxacin	Neurotoxicity and behavioral effects (10 µm > 0.1 µm > 1 µm microplastics); disrupted neural function (cell adhesion molecule pathway); levofloxacin mitigated dysbiosis with 1 µm microplastics	[83]
Avian	Muscovy duck	Polystyrene microplastics + chlortetracycline	Microplastics decreased chlortetracycline accumulation in liver/intestines, increased fecal excretion; microplastics caused oxidative stress, inflammation, gut barrier damage; co-exposure modulated gut microbiome, partially mitigating intestinal damage	[84]
Earthworm	*Eisenia fetida*	Polyethylene microplastics (13 µm, 48 µm, 150 µm) + tebuconazole	Greatest accumulation with 13 µm microplastics; increased oxidative stress markers; DNA damage and high toxicity	[85]
	*Eisenia fetida*	Polyethylene nanoplastics + pyraclostrobin	Extended persistence of pyraclostrobin in soil (+13 days); increased accumulation in worms (+8.4%); decreased body weight (–26.8%); altered gut microbiota; increased ARG diversity and plasmid-associated ARGs	[86]
	*Eisenia fetida*	Aged/UV-aged microplastics + azoxystrobin	Aged microplastics had higher adsorption/desorption; UV-aged microplastics + pesticide had lowest LC_50_ (highest toxicity); caused oxidative stress, skin and intestinal damage, impaired digestion; toxicity linked to pesticide desorption	[87]
	*Eisenia fetida*	Polystyrene microplastics + dufulin	Microplastics enhanced dufulin accumulation; oxidative stress at earlier stage (day 7 vs. day 14 for dufulin alone); altered 21 metabolites, disrupted 3 pathways (vs. 14 metabolites, 2 pathways for dufulin alone)	[88]
	*Enchytraeus crypticus*	Polyamide microplastics/polyvinyl chloride microplastics + tetracycline	Increased tetracycline accumulation; microplastics + tetracycline did not further enhance accumulation compared to tetracycline alone; decreased microbiota diversity; increased ARG diversity	[89]

### 4.6. Implications

The selection of pharmaceuticals and endpoints across aquatic and terrestrial models was primarily guided by environmental relevance, toxicological significance, and mechanistic value. Commonly used antibiotics such as sulfamethoxazole, tetracycline, doxycycline, chlortetracycline, and levofloxacin were studied due to their widespread use, frequent detection in wastewater, and co-occurrence with microplastics [81,84,89,90]. Agricultural pesticides and fungicides, including epoxiconazole, tebuconazole, pyraclostrobin, azoxystrobin, and dufulin, were prioritized in soil organisms because of their persistence and ecological importance [80,85,86,88]. Endpoints varied with study focus: acute studies often measured mortality, whereas chronic studies assessed organ-specific toxicity, oxidative stress, inflammation, endocrine disruption, developmental outcomes, and multigenerational effects [69]. Microbial endpoints, such as gut microbiota shifts, ARG enrichment, and dysbiosis, capture both ecological risks and public health implications [79,91]. The selection of experimental animals is typically driven by environmental relevance, exposure pathways, model accessibility, and established test guidelines, as well as sensitivity to toxicants. Rodents are commonly used as mammalian surrogates in human health risk assessment and standardized terrestrial toxicity testing, whereas zebrafish serve as a key model organism for ecotoxicity studies in aquatic environments [92,93]. Amphibians are sensitive indicators of endocrine and developmental disruption, making them ideal for testing thyroid axis and neurodevelopmental impacts [94].

The studies summarized indicate that co-exposure to microplastics and antimicrobials can lead to synergistic toxic effects across multiple terrestrial animal models. In rodent models, for example, findings revealed that microplastics facilitate the accumulation of antimicrobials (such as sulfamethoxazole and epoxiconazole) in key detoxifying organs like the liver and kidneys [76,79]. This accumulation is associated with enhanced oxidative stress, inflammation, and impaired metabolic function, as evidenced by increased malonaldehyde and NF-κβ levels coupled with a decline in antioxidant enzyme activity. Furthermore, early-life dietary exposure in juvenile mice demonstrated severe intestinal barrier disruption and adverse shifts in the gut microbiota [69,79], which suggest a heightened risk for microbial dysbiosis and the spread of ARGs.

For amphibians, the research highlights that co-exposure not only exacerbates neurotoxicity and developmental inhibition through mechanisms such as the disruption of cell adhesion molecule signaling but also leads to endocrine imbalances. Particularly, the capacity of certain microplastic particle sizes to cross the blood–brain barrier and interact with antibiotics like levofloxacin underscores their potential to alter normal growth and hormonal functions [82,83].

In earthworm models, the data reveal that microplastics enhance the bioaccumulation of pesticides and fungicides, intensifying oxidative stress and triggering metabolic disturbances [85,86,88]. These effects extend to changes in the composition and diversity of gut microbes, including an increase in ARG diversity and complexity, a finding that may have broader ecological implications given the role of earthworms in soil health and nutrient cycling.

A study on enchytraeids also signals significant microbiome disruptions and a rise in ARG diversity upon co-exposure to antimicrobials and microplastics, though it does not establish synergistic toxicity [89]. However, microplastics and chlortetracycline were found to mitigate some intestinal damage in ducks by modulating the gut microbiome [84]. Collectively, these implications suggest that environmental exposures to these combined pollutants could have adverse consequences not only for individual organisms but also for entire ecosystems, by altering physiological processes and microbial communities essential for health.

The studies across rodents, amphibians, birds, and soil invertebrates reveal a fairly universal pattern of combined toxicity between microplastics and antimicrobials in terrestrial ecosystems. Co-exposure generally increases pollutant accumulation in tissues, exacerbates oxidative stress and inflammation, disrupts gut or intestinal barrier integrity, and alters microbiota composition in ways that promote the spread of ARGs. Microplastics often act as carriers that enhance the bioavailability and persistence of antimicrobials, while simultaneously impairing detoxification and metabolic processes. The result is synergistic or at least additive toxicity, manifested in organ damage, immune dysregulation, developmental inhibition, and even neurotoxicity. Although the magnitude of effects can vary with polymer type, particle size, or aging state, the consistent theme is that microplastics intensify the biological impacts of antimicrobials through combined mechanisms of physical stress, altered pharmacokinetics, and microbiome disruption.

### 4.7. Limitations and Future Directions

Despite important insights, these studies have limitations. Most experiments were conducted in controlled laboratory settings with relatively short exposure durations, which may not fully reflect chronic environmental conditions. Dose levels and particle sizes often vary from real-world exposure, and the combined effects of microplastics and antimicrobials may differ in more complex ecosystems. Additionally, although some molecular pathways were identified (e.g., Nrf2 signaling, tricarboxylic acid cycle disruptions), many mechanistic details remain unclear, limiting a comprehensive understanding of how these pollutants interact at the cellular level.

Across taxa, co-exposure to microplastics and antimicrobials consistently drives oxidative stress, inflammatory responses, gut microbiota disruption, barrier impairment, and enhanced pollutant accumulation, pointing to a shared core of interactive toxicity. However, species- and context-specific mechanisms shape distinct outcomes: rodents show gut–brain axis and molecular signaling disruption, amphibians display thyroid axis interference and blood–brain barrier penetration, earthworms exhibit strong soil-mediated chemical persistence and ARG shifts, and birds sometimes experience altered pharmacokinetics that mitigate rather than amplify antibiotic toxicity. These contrasts suggest that while the underlying toxicological themes are convergent, extrapolation across taxa requires caution, as organismal physiology and environmental context modulate how these shared mechanisms manifest.

Furthermore, studies on the combined toxicity of microplastics and antimicrobials on terrestrial animals are much fewer than those on aquatic animals and are limited to a few species, especially mice, frogs, and earthworms. Studies on antibiotics are disproportionately higher than those on other antimicrobials.

Future research should prioritize long-term, multigenerational studies using environmentally relevant concentrations and real-world conditions. For instance, studies can be conducted on pregnant or gravid organisms, specifically rodents, amphibians, and birds, to examine the combined toxicity of microplastics and antimicrobials at environmentally relevant concentrations on the parents and offspring. Incorporating real-world environmental dynamics such as particle aging, dilution, trophic transfer, and microbial degradation is essential to improve ecological realism. For instance, using environmentally sourced or specially aged, polydisperse microplastics can better reflect natural conditions. In addition, investigating how organisms at higher trophic levels are exposed through ingesting contaminated prey would provide critical insights into the trophic transfer of microplastics and antimicrobials, as well as their cumulative toxic effects. Field-based experiments incorporating multiple stressors (e.g., pesticides, heavy metals, temperature fluctuations) would improve ecological relevance. Employing omics techniques can reveal deeper mechanistic insights and help identify early biomarkers of combined toxicity. Moreover, integrated risk assessments and interdisciplinary approaches will be essential for developing effective pollution mitigation strategies and protecting terrestrial ecosystems from the compounded threats posed by microplastics and antimicrobials. These combined toxicity studies should be expanded to include more terrestrial species to gain a better understanding of how different species are affected by the presence of microplastics and antimicrobials in the environment. Further research on other antimicrobials, like antifungals and antivirals, is needed.

## 5. Brief Overview of Influences of Microplastic Properties on Combined Toxicity

This section provides a brief overview of how microplastic properties affect their combined toxicity with antibiotics. The content is not discussed in detail as this is not the main scope of this review. Microplastic properties, such as size and aging, significantly influence their combined toxicity with antibiotics in aquatic animals by altering adsorption capacity, bioavailability, and physiological interactions. Smaller microplastics (e.g., 1 µm) generally exhibit higher surface area and greater adsorption of antibiotics compared to larger particles (e.g., 10 µm), which can modify the concentration of freely available antibiotics and subsequently affect toxicity. For instance, Zhang et al. [22] reported that co-exposure of 1 µm polystyrene microplastics with roxithromycin reduced oxidative stress biomarkers compared to roxithromycin alone, while 10 µm particles showed different enzyme activity responses, suggesting size-dependent interactions. Aging processes, such as UV exposure, further enhance microplastic surface oxidation and functional groups, increasing their ability to adsorb antibiotics and modify biological effects. Liu et al. [41] demonstrated that UV-aged polystyrene microplastics improved survival and mitigated reproductive toxicity in *Daphnia magna* under roxithromycin exposure, but intensified oxidative damage in the next generation, indicating that aging influences both immediate and intergenerational toxicity. Additionally, aged microplastics can facilitate higher antibiotic accumulation in fish tissues, as shown by Zhang et al. [57], where smaller aged particles amplified roxithromycin-induced organ damage and gut microbiota shifts more than larger ones.

Similar observations have been reported in terrestrial animals. Smaller microplastics possess a higher surface area and more reactive sites, enabling stronger adsorption of antibiotics, which facilitates their co-transport and accumulation in critical organs like the liver and kidneys, as shown in mice exposed to sulfamethoxazole and polystyrene microplastics [76]. This adsorption enhances oxidative stress and inflammation through pathways such as Keap1–Nrf2, leading to tissue damage and metabolic disruption [76]. Additionally, microplastics can impair gut barrier integrity and alter gut microbiota composition, promoting dysbiosis, proliferation of antibiotic-resistant bacteria, and dissemination of resistance genes, as observed in juvenile mice exposed to tetracycline or doxycycline [79,81]. Aging further amplifies toxicity by introducing oxygen-containing functional groups on microplastic surfaces, increasing their ability to adsorb and release co-contaminants, as seen in earthworms, where aged microplastics enhanced fungicide toxicity and oxidative stress [87]. Moreover, microplastic size influences the extent of pollutant accumulation and physiological interference; for instance, smaller polyethylene particles increased fungicide retention and gut microbiota shifts in earthworms more than larger particles [85]. While the effects of microplastic size on combined toxicity were less consistent in amphibians, it was observed that smaller microplastics could cross the blood–brain barrier [82].

Overall, microplastic properties such as size and aging play a critical role in modulating their combined toxicity with antibiotics. Smaller particles, due to their higher surface area and reactivity, exhibit stronger antibiotic adsorption and co-transport, leading to increased bioaccumulation, oxidative stress, and gut microbiota disruption. Aging generally enhances these interactions by introducing oxygen-containing functional groups, increasing sorption capacity, and influencing both immediate and intergenerational toxicity, though it was reported to mitigate toxicity in some cases.

## 6. Conclusions

The mounting body of research on the combined toxicity of microplastics and antimicrobials underscores a complex and multifaceted environmental hazard affecting both aquatic and terrestrial ecosystems. Microplastics not only serve as physical stressors but also function as carriers and modulators of antimicrobials, such as antibiotics, antifungals, and antivirals. Their interactions frequently result in enhanced bioavailability, altered toxicokinetics, and amplified biological effects, including oxidative stress, apoptosis, neurotoxicity, and developmental disruption, in a wide range of organisms. Notably, aged or surface-functionalized microplastics exhibit stronger interactions with these chemicals, intensifying the delivery and toxicity of co-contaminants. These synergistic effects can compromise organismal health, damage key physiological systems (e.g., liver, intestines, nervous and cardiovascular systems), and potentially threaten population stability and ecosystem functions.

From a practical standpoint, these findings hold significant implications for environmental monitoring, risk assessment, and pollutant management. Regulatory frameworks must account for the co-occurrence and interactive effects of microplastics and antimicrobials, rather than evaluating contaminants in isolation. This includes establishing standards and monitoring the levels of microplastics and antimicrobials in wastewater, especially from hospital and pharmaceutical sources. The design of future toxicity tests, mitigation strategies, and pollution thresholds should integrate the potential for microplastic-facilitated transport and transformation of chemical agents. This review resonates with the call for a harmonized ecotoxicological effect and risk assessment framework for microplastics [95]. Moreover, industries involved in agriculture, aquaculture, and wastewater treatment should be aware of how plastic debris may interact with antimicrobial residues, potentially prolonging environmental persistence and toxicity.

Looking ahead, research must shift toward more ecologically relevant and mechanistically informative approaches. This includes the use of environmentally representative concentrations, aged and heterogeneous microplastics, complex pollutant mixtures, and diverse test species across life stages and trophic levels. Advanced molecular tools, such as transcriptomics, metabolomics, and microbiome analyses, can offer deeper insights into the underlying biological pathways and adaptive responses. Long-term and multigenerational studies are essential for assessing cumulative and evolutionary effects, while investigations of trophic transfer and food web dynamics are crucial for understanding ecosystem-wide consequences. Current research primarily focuses on the interactions between microplastics and antibiotics, highlighting the importance of including a wider range of antivirals, antifungals, and other antimicrobials in future studies.

In summary, the interactions between microplastics and antimicrobials pose a pressing environmental challenge that necessitates interdisciplinary collaboration, methodological innovation, and proactive policy responses to safeguard both environmental and public health.

## Data Availability

This review paper does not report original data. All data presented or discussed are derived from previously published studies, which are cited throughout the manuscript. No new datasets were generated or analyzed for this study.

## Supplementary Materials

The following supporting information can be downloaded at: https://www.mdpi.com/article/10.3390/antibiotics14090896/s1, Table S1: Keywords used for papers included in full-text review.
