# Supplementary material for: Combined Toxicity of Microplastics and Antimicrobials on Animals: A Review
Source: Antibiotics (Basel). 2025 Sep 5;14(9):896. doi: 10.3390/antibiotics14090896 (PMC12466353; doi:10.3390/antibiotics14090896)
Supplement: Supplementary file 1 [file antibiotics-14-00896-s001.zip › antibiotics-3818535-supplementary.pdf]

Paper	Keywords
1. WasteDirect. Plastic Waste Statistics & Trends. Available online: https://wastedirect.co.uk/blog/plastic-waste-statistics/ (accessed on 22 July).	plastic, microplastic
2. Ritchie, H.; Samborska, V.; Roser, M. Plastic Pollution. 2023.	plastic pollution
3. Tang, K.H.D. Microplastics and Antibiotics in Aquatic Environments: A Review of Their Interactions and Ecotoxicological Implications. Tropical Aquatic and Soil Pollution 2024, 4, 60-78.	microplastics, antibiotics, antimicrobials, co-exposure, toxicity, aquatic organisms
4. Tang, K.H.; Li, R. Aged Microplastics and Antibiotic Resistance Genes: A Review of Aging Effects on Their Interactions. Antibiotics 2024, 13.	microplastics, antibiotics, antimicrobials, co-exposure, aquatic organisms
5. Crew, A.; Gregory-Eaves, I.; Ricciardi, A. Distribution, abundance, and diversity of microplastics in the upper St. Lawrence River. Environmental Pollution 2020, 260, 113994.	microplastics, aquatic organisms, toxicity
6. Hermsen, E.; Pompe, R.; Besseling, E.; Koelmans, A.A. Detection of low numbers of microplastics in North Sea fish using strict quality assurance criteria. Marine Pollution Bulletin 2017, 122, 253-258.	microplastics, fish, toxicity
7. Au, S.Y.; Lee, C.M.; Weinstein, J.E.; van den Hurk, P.; Klaine, S.J. Trophic transfer of microplastics in aquatic ecosystems: Identifying critical research needs. Integrated Environmental Assessment and Management 2017, 13, 505-509.	microplastics, aquatic organisms, co-exposure, toxicity
8. Tang, K.H.D.; Zhou, J. Ecotoxicity of Biodegradable Microplastics and Bio-based Microplastics: A Review of in vitro and in vivo Studies. Environmental Management 2024.	microplastics, co-exposure, toxicity, aquatic organisms, animals
9. Ateia, M.; Zheng, T.; Calace, S.; Tharayil, N.; Pilla, S.; Karanfil, T. Sorption behavior of real microplastics (MPs): Insights for organic micropollutants adsorption on a large set of well-characterized MPs. Science of The Total Environment 2020, 720, 137634.	microplastics, antimicrobials, antibiotics, co-exposure
10. Chen, Y.; Li, J.; Wang, F.; Yang, H.; Liu, L. Adsorption of tetracyclines onto polyethylene microplastics: A combined study of experiment and molecular dynamics simulation. Chemosphere 2021, 265, 129133.	microplastics, tetracycline, co-exposure, aquatic organisms, toxicity
11. Wei, X.; Li, M.; Wang, Y.; Jin, L.; Ma, G.; Yu, H. Developing Predictive Models for Carrying Ability of Micro-Plastics towards Organic Pollutants. Molecules 2019, 24.	microplastics, antimicrobials, co-exposure
12. Zhang, M.; Xu, L. Transport of micro- and nanoplastics in the environment: Trojan-Horse effect for organic contaminants. Critical Reviews in Environmental Science and Technology 2022, 52, 810-846.	microplastics, antimicrobials, co-exposure

Paper	Keywords
13. Koelmans, A.A.; Bakir, A.; Burton, G.A.; Janssen, C.R. Microplastic as a Vector for Chemicals in the Aquatic Environment: Critical Review and Model-Supported Reinterpretation of Empirical Studies. Environmental Science & Technology 2016, 50, 3315-3326.	microplastics, antimicrobials, co-exposure, aquatic organisms
14. Tang, K.H.D. Environmental Co-existence of Microplastics and Perfluorochemicals: A Review of Their Interactions. Biointerface Research in Applied Chemistry 2023, 13, 587.	microplastics, co-exposure, aquatic organisms
15. Yuan, F.; Chen, H.; Ding, Y.; Wang, Y.; Liao, Q.; Wang, T.; Fan, Q.; Feng, Z.; Zhang, C.; Fu, G.; et al. Effects of microplastics on the toxicity of co-existing pollutants to fish: A meta-analysis. Water Research 2023, 240, 120113.	microplastics, co-exposure, fish, toxicity
16. Rainieri, S.; Conlledo, N.; Larsen, B.K.; Granby, K.; Barranco, A. Combined effects of microplastics and chemical contaminants on the organ toxicity of zebrafish (Danio rerio). Environmental Research 2018, 162, 135-143.	microplastics, co-exposure, fish, toxicity, combined toxicity
17. Chen, Q.; Yin, D.; Jia, Y.; Schiwy, S.; Legradi, J.; Yang, S.; Hollert, H. Enhanced uptake of BPA in the presence of nanoplastics can lead to neurotoxic effects in adult zebrafish. Science of The Total Environment 2017, 609, 1312-1321.	microplastics, co-exposure, fish, toxicity
18. Liu, Y.; Zhang, J.; Zhao, H.; Cai, J.; Sultan, Y.; Fang, H.; Zhang, B.; Ma, J. Effects of polyvinyl chloride microplastics on reproduction, oxidative stress and reproduction and detoxification-related genes in Daphnia magna. Comparative Biochemistry and Physiology Part C: Toxicology & Pharmacology 2022, 254, 109269.	microplastics, co-exposure, invertebrates, toxicity
19. Lu, J.; Wu, J.; Gong, L.; Cheng, Y.; Yuan, Q.; He, Y. Combined toxicity of polystyrene microplastics and sulfamethoxazole on zebrafish embryos. Environmental Science and Pollution Research 2022, 29, 19273-19282.	microplastics, sulfamethoxazole, combined toxicity, fish, co-exposure, toxicity
20. Li, N.; Zeng, Z.; Zhang, Y.; Zhang, H.; Tang, N.; Guo, Y.; Lu, L.; Li, X.; Zhu, Z.; Gao, X.; et al. Higher toxicity induced by co-exposure of polystyrene microplastics and chloramphenicol to Microcystis aeruginosa: Experimental study and molecular dynamics simulation. Science of The Total Environment 2023, 866, 161375.	microplastics, chloramphenicol, co-exposure, combined toxicity
21. Zhang, S.; Ding, J.; Razanajatovo, R.M.; Jiang, H.; Zou, H.; Zhu, W. Interactive effects of polystyrene microplastics and roxithromycin on bioaccumulation and biochemical status in the freshwater fish red tilapia (Oreochromis niloticus). Science of The Total Environment 2019, 648, 1431-1439.	microplastics, roxithromycin, co-exposure, fish, toxicity, combined toxicity
22. Zhang, P.; Yan, Z.; Lu, G.; Ji, Y. Single and combined effects of microplastics and roxithromycin on Daphnia magna. Environmental Science and Pollution Research 2019, 26, 17010-17020.	microplastics, roxithromycin, co-exposure, invertebrates, combined toxicity

Paper	Keywords
23. Tang, K.H.D. Abundance of microplastics in wastewater treatment sludge. Journal of Human, Earth, and Future 2022, 3, 138-146.	microplastics, co-exposure, aquatic organisms
24. Löffler, P.; Escher, B.I.; Baduel, C.; Virta, M.P.; Lai, F.Y. Antimicrobial Transformation Products in the Aquatic Environment: Global Occurrence, Ecotoxicological Risks, and Potential of Antibiotic Resistance. Environmental Science & Technology 2023, 57, 9474-9494.	antimicrobials, antibiotics, co-exposure, aquatic organisms, toxicity
25. Prajapati, A.; Narayan Vaidya, A.; Kumar, A.R. Microplastic properties and their interaction with hydrophobic organic contaminants: a review. Environmental Science and Pollution Research 2022, 29, 49490-49512.	microplastics, antimicrobials, co-exposure, toxicity
26. Kovalakova, P.; Cizmas, L.; McDonald, T.J.; Marsalek, B.; Feng, M.; Sharma, V.K. Occurrence and toxicity of antibiotics in the aquatic environment: A review. Chemosphere 2020, 251, 126351.	antibiotics, co-exposure, aquatic organisms, toxicity
27. Tang, K.H. Terrestrial and Aquatic Plastisphere: Formation, Characteristics, and Influencing Factors. Sustainability 2024, 16.	microplastics, co-exposure, aquatic organisms
28. Naghavi, M.; Vollset, S.E.; Ikuta, K.S.; Swetschinski, L.R.; Gray, A.P.; Wool, E.E.; Robles Aguilar, G.; Mestrovic, T.; Smith, G.; Han, C.; et al. Global burden of bacterial antimicrobial resistance 1990–2021: a systematic analysis with forecasts to 2050. The Lancet 2024, 404, 1199-1226.	antimicrobials, antibiotics, co-exposure, toxicity
29. Lai, K.P.; Tsang, C.F.; Li, L.; Yu, R.M.K.; Kong, R.Y.C. Microplastics act as a carrier for wastewater-borne pathogenic bacteria in sewage. Chemosphere 2022, 301, 134692.	microplastics, antimicrobials, co-exposure
30. Cholewińska, P.; Moniuszko, H.; Wojnarowski, K.; Pokorny, P.; Szeligowska, N.; Dobicki, W.; Polechoński, R.; Górniak, W. The Occurrence of Microplastics and the Formation of Biofilms by Pathogenic and Opportunistic Bacteria as Threats in Aquaculture. International Journal of Environmental Research and Public Health 2022, 19.	microplastics, antimicrobials, co-exposure, aquatic organisms
31. Su, H.; Xu, W.; Hu, X.; Xu, Y.; Wen, G.; Cao, Y. The impact of microplastics on antibiotic resistance genes, metal resistance genes, and bacterial community in aquaculture environment. Journal of Hazardous Materials 2025, 489, 137704.	microplastics, antibiotics, co-exposure, aquatic organisms, toxicity
32. Zhuang, S.; Wang, J. Interaction between antibiotics and microplastics: Recent advances and perspective. Science of The Total Environment 2023, 897, 165414.	microplastics, antibiotics, co-exposure, toxicity
33. Zhang, Y.; Xu, X.; Xu, J.; Li, Z.; Cheng, L.; Fu, J.; Sun, W.; Dang, C. When antibiotics encounter microplastics in aquatic environments: Interaction, combined toxicity, and risk assessments. Science of The Total Environment 2024, 929, 172455.	microplastics, antibiotics, combined toxicity, co-exposure,

Paper	Keywords
	aquatic organisms, toxicity
34. Yu, Z.; An, Q.; Zhou, T.; Zhou, L.; Yan, B. Meta-analysis unravels the complex combined toxicity of microplastics and antibiotics in aquatic ecosystems. Science of The Total Environment 2024, 929, 172503.	microplastics, antibiotics, combined toxicity, co-exposure, aquatic organisms, toxicity
35. Wang, Y.; Yang, Y.; Liu, X.; Zhao, J.; Liu, R.; Xing, B. Interaction of Microplastics with Antibiotics in Aquatic Environment: Distribution, Adsorption, and Toxicity. Environmental Science & Technology 2021, 55, 15579-15595.	microplastics, antibiotics, co-exposure, aquatic organisms, toxicity
36. Wang, K.; Guo, C.; Li, J.; Wang, K.; Liang, S.; Wang, W.; Wang, J. A critical review of the adsorption-desorption characteristics of antibiotics on microplastics and their combined toxic effects. Environmental Technology & Innovation 2024, 35, 103729.	microplastics, antibiotics, combined toxicity, co-exposure, aquatic organisms, toxicity
37. Wei, J.; Chen, M.; Wang, J. Insight into combined pollution of antibiotics and microplastics in aquatic and soil environment: Environmental behavior, interaction mechanism and associated impact of resistant genes. TrAC Trends in Analytical Chemistry 2023, 166, 117214.	microplastics, antibiotics, co-exposure, toxicity
38. Zhang, X.; Liu, L.; Chen, X.; Li, J.; Chen, J.; Wang, H. The fate and risk of microplastic and antibiotic sulfamethoxazole coexisting in the environment. Environmental Geochemistry and Health 2023, 45, 2905-2915.	microplastics, sulfamethoxazole, co-exposure, aquatic organisms, toxicity
39. Feng, L.-J.; Zhang, K.-X.; Shi, Z.-L.; Zhu, F.-P.; Yuan, X.-Z.; Zong, W.-S.; Song, C. Aged microplastics enhance their interaction with ciprofloxacin and joint toxicity on Escherichia coli. Ecotoxicology and Environmental Safety 2022, 247, 114218.	microplastics, ciprofloxacin, combined toxicity, co-exposure, toxicity
40. He, S.; Tong, J.; Xiong, W.; Xiang, Y.; Peng, H.; Wang, W.; Yang, Y.; Ye, Y.; Hu, M.; Yang, Z.; et al. Microplastics influence the fate of antibiotics in freshwater environments: Biofilm formation and its effect on adsorption behavior. Journal of Hazardous Materials 2023, 442, 130078.	microplastics, antibiotics, co-exposure, aquatic organisms, toxicity
41. Liu, J.; Yang, H.; Meng, Q.; Feng, Q.; Yan, Z.; Liu, J.; Liu, Z.; Zhou, Z. Intergenerational and biological effects of roxithromycin and polystyrene microplastics to Daphnia magna. Aquatic Toxicology 2022, 248, 106192.	microplastics, roxithromycin, co-exposure, invertebrates, toxicity
42. Yin, C.; Yang, X.; Zhao, T.; Watson, P.; Yang, F.; Liu, H. Changes of the acute and chronic toxicity of three antimicrobial agents to Daphnia magna in the presence/absence of micro-polystyrene. Environmental Pollution 2020, 263, 114551.	microplastics, antibiotics, antifungals, antivirals, co-exposure, invertebrates, toxicity

Paper	Keywords
43. Nugnes, R.; Russo, C.; Lavorgna, M.; Orlo, E.; Kundi, M.; Isidori, M. Polystyrene microplastic particles in combination with pesticides and antiviral drugs: Toxicity and genotoxicity in Ceriodaphnia dubia. Environmental Pollution 2022, 313, 120088.	microplastics, antivirals, combined toxicity, co-exposure, invertebrates, toxicity
44. Said, R.E.M.; Hamed, M.; Shaalan, W.M.; Elbaghdady, H.A.M.; Sayed, A.E.-D.H. Exploring the Coexposure Effects of Pyrogallol and Microplastic on the Red Swamp Crayfish Procambarus clarkii. Aquaculture Research 2025, 6084150.	microplastics, co-exposure, invertebrates, toxicity
45. Han, Y.; Zhou, W.; Tang, Y.; Shi, W.; Shao, Y.; Ren, P.; Zhang, J.; Xiao, G.; Sun, H.; Liu, G. Microplastics aggravate the bioaccumulation of three veterinary antibiotics in the thick shell mussel Mytilus coruscus and induce synergistic immunotoxic effects. Science of The Total Environment 2021, 770, 145273.	microplastics, antibiotics, combined toxicity, co-exposure, aquatic organisms, toxicity
46. Guo, X.; Cai, Y.; Ma, C.; Han, L.; Yang, Z. Combined toxicity of micro/nano scale polystyrene plastics and ciprofloxacin to Corbicula fluminea in freshwater sediments. Science of The Total Environment 2021, 789, 147887.	microplastics, ciprofloxacin, combined toxicity, co-exposure, aquatic organisms, toxicity
47. Zhou, W.; Tang, Y.; Du, X.; Han, Y.; Shi, W.; Sun, S.; Zhang, W.; Zheng, H.; Liu, G. Fine polystyrene microplastics render immune responses more vulnerable to two veterinary antibiotics in a bivalve species. Marine Pollution Bulletin 2021, 164, 111995.	microplastics, antibiotics, co-exposure, aquatic organisms, combined toxicity
48. Ma, Y.; Gao, Y.; Xu, R.; Li, D.; Waiho, K.; Wang, Y.; Hu, M. Combined toxic effects of nanoplastics and norfloxacin on antioxidant and immune genes in mussels. Marine Environmental Research 2024, 193, 106277.	microplastics, norfloxacin, combined toxicity, co-exposure, aquatic organisms, toxicity
49. Zhang, P.; Lu, G.; Sun, Y.; Yan, Z.; Zhang, L.; Liu, J. Effect of microplastics on oxytetracycline trophic transfer: Immune, gut microbiota and antibiotic resistance gene responses. Journal of Hazardous Materials 2024, 470, 134147.	microplastics, oxytetracycline, co-exposure, aquatic organisms, toxicity
50. Chen, J.; Lei, Y.; Wen, J.; Zheng, Y.; Gan, X.; Liang, Q.; Huang, C.; Song, Y. The neurodevelopmental toxicity induced by combined exposure of nanoplastics and penicillin in embryonic zebrafish: The role of aging processes. Environmental Pollution 2023, 335, 122281.	microplastics, penicillin, combined toxicity, co-exposure, fish, toxicity
51. Zhang, J.; Bai, Y.; Meng, H.; Zhu, Y.; Yue, H.; Li, B.; Wang, J.; Wang, J.; Zhu, L.; Du, Z. Combined toxic effects of polystyrene microplastics and 3,6-dibromocarbazole on zebrafish (Danio rerio) embryos. Science of The Total Environment 2024, 913, 169787.	microplastics, combined toxicity, co-exposure, fish, toxicity
52. Li, C.; Yuan, S.; Zhou, Y.; Li, X.; Duan, L.; Huang, L.; Zhou, X.; Ma, Y.; Pang, S. Microplastics reduce the bioaccumulation and	microplastics, antifungals, co-exposure, fish, toxicity

Paper	Keywords
oxidative stress damage of triazole fungicides in fish. Science of The Total Environment 2022, 806, 151475.	
53. Bhagat, J.; Zang, L.; Nakayama, H.; Nishimura, N.; Shimada, Y. Effects of nanoplastic on toxicity of azole fungicides (ketoconazole and fluconazole) in zebrafish embryos. Science of The Total Environment 2021, 800, 149463.	microplastics, antifungals, co-exposure, fish, toxicity
54. Bhagat, J.; Nishimura, N.; Shimada, Y. Toxicological interactions of microplastics/nanoplastics and environmental contaminants: Current knowledge and future perspectives. Journal of Hazardous Materials 2021, 405, 123913.	microplastics, antimicrobials, antibiotics, antifungals, antivirals, co-exposure, aquatic organisms, toxicity
55. Wu, Y.; Zhu, Z.; Zhong, R.; Fang, X.; Wang, X.; Huang, Y.; Gong, H.; Yan, M. Microplastics Enhance the Toxic Effects of Tetracycline on the Early Development of Zebrafish in a Dose-Dependent Manner. Fishes 2025, 10.	microplastics, tetracycline, combined toxicity, co-exposure, fish, toxicity
56. Xiong, G.; Zhang, H.; Shi, H.; Peng, Y.; Han, M.; Hu, T.; Liao, X.; Liu, Y.; Zhang, J.e.; Xu, G. Enhanced hepatotoxicity in zebrafish due to co-exposure of microplastics and sulfamethoxazole: Insights into ROS-mediated MAPK signaling pathway regulation. Ecotoxicology and Environmental Safety 2024, 278, 116415.	microplastics, sulfamethoxazole, co-exposure, fish, toxicity
57. Zhang, P.; Lu, G.; Sun, Y.; Zhang, J.; Liu, J.; Yan, Z. Aged microplastics change the toxicological mechanism of roxithromycin on Carassius auratus: Size-dependent interaction and potential long-term effects. Environment International 2022, 169, 107540.	microplastics, roxithromycin, co-exposure, fish, toxicity
58. Juan, K.; Boya, F.; Julin, Y.; Liqin, Y. Combined Effects of Environmental Concentration of Oxytetracycline and Polystyrene Microplastics on Intestinal Tract of Juvenile Yellow Catfish (Pelteobagrus fulvidraco). Asian Journals of Ecotoxicology 2023, 18.	microplastics, oxytetracycline, co-exposure, fish, toxicity
59. Fonte, E.; Ferreira, P.; Guilhermino, L. Temperature rise and microplastics interact with the toxicity of the antibiotic cefalexin to juveniles of the common goby (Pomatoschistus microps): Post-exposure predatory behaviour, acetylcholinesterase activity and lipid peroxidation. Aquatic Toxicology 2016, 180, 173-185.	microplastics, cefalexin, co-exposure, fish, toxicity
60. Zhang, Y.T.; Gouveia, A.; Chen, R.; Xing, D.; Wang, J.; Mu, J. Biomicroplastics and Antibiotics: A Toxic Cocktail for Fatty Liver Disease in Marine Medaka. Environmental Science & Technology 2025, 59, 12485-12494.	microplastics, antibiotics, co-exposure, fish, toxicity
61. Zhao, P.; Lu, W.; Avellán-Llaguno, R.D.; Liao, X.; Ye, G.; Pan, Z.; Hu, A.; Huang, Q. Gut microbiota related response of Oryzias melastigma to combined exposure of polystyrene microplastics and tetracycline. Science of The Total Environment 2023, 905, 167359.	microplastics, tetracycline, co-exposure, fish, toxicity

Paper	Keywords
62. Wu, X.; Zhang, X.; Liao, H.; Guo, J.; Ma, Z.; Fu, Z. Microplastics and tetracycline affecting apoptosis, enzyme activities and metabolism processes in the Aurelia aurita polyps: insights into combined pollutant effects. Frontiers in Marine Science 2025, 12.	microplastics, tetracycline, combined toxicity, co-exposure, aquatic organisms, toxicity
63. Huang, M.; Ma, Y.; Qian, J.; Sokolova, I.M.; Zhang, C.; Waiho, K.; Fang, J.K.H.; Ma, X.; Wang, Y.; Hu, M. Combined effects of norfloxacin and polystyrene nanoparticles on the oxidative stress and gut health of the juvenile horseshoe crab Tachypleus tridentatus. Journal of Hazardous Materials 2024, 468, 133801.	microplastics, norfloxacin, combined toxicity, co-exposure, aquatic organisms, toxicity
64. Kinigopoulou, V.; Pashalidis, I.; Kalderis, D.; Anastopoulos, I. Microplastics as carriers of inorganic and organic contaminants in the environment: A review of recent progress. Journal of Molecular Liquids 2022, 350, 118580.	microplastics, co-exposure, toxicity
65. Dey, S.; Rout, A.K.; Ghosh, K.; Jana, A.K.; Behera, B.K. Microbial Ecology in Microplastics: Impact on Aquatic Ecosystems and Bioremediation. In Current Trends in Fisheries Biotechnology, Behera, B.K., Ed.; Springer Nature Singapore: Singapore, 2024; pp. 79-93.	microplastics, co-exposure, aquatic organisms, toxicity
66. Liu, P.; Dai, J.; Bie, C.; Li, H.; Zhang, Z.; Guo, X.; Zhu, L. Bioaccessibility of Microplastic-Associated Antibiotics in Freshwater Organisms: Highlighting the Impacts of Biofilm Colonization via an In Vitro Protocol. Environmental Science & Technology 2022, 56, 12267-12277.	microplastics, antibiotics, co-exposure, aquatic organisms, toxicity
67. Ju, H.; Yang, X.; Osman, R.; Geissen, V. The role of microplastic aging on chlorpyrifos adsorption-desorption and microplastic bioconcentration. Environmental Pollution 2023, 331, 121910.	microplastics, co-exposure, toxicity
68. Hu, Q.; Wang, H.; He, C.; Jin, Y.; Fu, Z. Polystyrene nanoparticles trigger the activation of p38 MAPK and apoptosis via inducing oxidative stress in zebrafish and macrophage cells. Environmental Pollution 2021, 269, 116075.	microplastics, co-exposure, fish, toxicity
69. Tang, K.H.D. A review of the toxic effects of microplastics based on studies on mammals and mammalian cell lines. Environmental Science: Advances 2024, 3, 1669-1678.	microplastics, co-exposure, mammals, toxicity
70. Cui, W.; Hale, R.C.; Huang, Y.; Zhou, F.; Wu, Y.; Liang, X.; Liu, Y.; Tan, H.; Chen, D. Sorption of representative organic contaminants on microplastics: Effects of chemical physicochemical properties, particle size, and biofilm presence. Ecotoxicology and Environmental Safety 2023, 251, 114533.	microplastics, co-exposure, toxicity
71. Du, J.; Zhan, L.; Zhang, G.; Zhou, Q.; Wu, W. Antibiotic sorption onto MPs in terrestrial environment: a critical review of the transport, bioaccumulation, ecotoxicological effects and prospects. Drug and Chemical Toxicology 2025, 48, 266-280.	microplastics, antibiotics, co-exposure, toxicity

Paper	Keywords
72. Ullah, F.; Wang, P.-Y.; Saqib, S.; Zhao, L.; Ashraf, M.; Khan, A.; Khan, W.; Khan, A.; Chen, Y.; Xiong, Y.-C. Toxicological complexity of microplastics in terrestrial ecosystems. iScience 2025, 28.	microplastics, co-exposure, toxicity
73. Tang, K.H.D. Microplastics in Soil: Uncovering Their Hidden Chemical Implications. Tropical Aquatic and Soil Pollution 2025, 5, 88-109.	microplastics, co-exposure, toxicity
74. Dissanayake, P.D.; Kim, S.; Sarkar, B.; Oleszczuk, P.; Sang, M.K.; Haque, M.N.; Ahn, J.H.; Bank, M.S.; Ok, Y.S. Effects of microplastics on the terrestrial environment: A critical review. Environmental Research 2022, 209, 112734.	microplastics, co-exposure, toxicity
75. Piergiacomo, F.; Brusetti, L.; Pagani, L. Understanding the Interplay between Antimicrobial Resistance, Microplastics and Xenobiotic Contaminants: A Leap towards One Health? International Journal of Environmental Research and Public Health 2023, 20.	microplastics, antibiotics, antifungals, antivirals, co-exposure, toxicity
76. Fu, J.; Zhang, L.; Xiang, K.; Zhang, Y.; Wang, G.; Chen, L. Microplastic-contaminated antibiotics as an emerging threat to mammalian liver: enhanced oxidative and inflammatory damages. Biomaterials Science 2023, 11, 4298-4307.	microplastics, antibiotics, mammals, co-exposure, toxicity
77. Xia, Y.; Lan, Y.; Xu, Y.; Liu, F.; Chen, X.; Luo, J.; Xu, H.; Liu, Y. Effects of microplastics and tetracycline induced intestinal damage, intestinal microbiota dysbiosis, and antibiotic resistome: metagenomic analysis in young mice. Environment International 2025, 199, 109512.	microplastics, tetracycline, co-exposure, mammals, toxicity
78. Sun, W.; Yan, S.; Meng, Z.; Tian, S.; Jia, M.; Huang, S.; Wang, Y.; Zhou, Z.; Diao, J.; Zhu, W. Combined ingestion of polystyrene microplastics and epoxiconazole increases health risk to mice: Based on their synergistic bioaccumulation in vivo. Environment International 2022, 166, 107391.	microplastics, antifungals, combined toxicity, co-exposure, mammals, toxicity
79. Sun, H.; Yang, B.; Zhu, X.; Li, Q.; Song, E.; Song, Y. Oral exposure of polystyrene microplastics and doxycycline affects mice neurological function via gut microbiota disruption: The orchestrating role of fecal microbiota transplantation. Journal of Hazardous Materials 2024, 467, 133714.	microplastics, doxycycline, co-exposure, mammals, toxicity
80. Zhang, W.; Teng, M.; Yan, J. Combined effect and mechanism of microplastic with different particle sizes and levofloxacin on developing Rana nigromaculata: Insights from thyroid axis regulation and immune system. Journal of Environmental Management 2024, 366, 121833.	microplastics, levofloxacin, combined toxicity, co-exposure, amphibians, toxicity
81. Zhang, W.; Zhang, W.; Teng, M.; Xu, J.; Wang, J.; Yang, J.; Liu, Y. The effect and mechanism of variable particle size microplastics and levofloxacin on the neurotoxicity of Rana nigromaculata based on	microplastics, levofloxacin, co-exposure, amphibians, toxicity

Paper	Keywords
the microorganism-intestine-brain axis. Journal of Environmental Management 2024, 354, 120329.	
82. Liu, B.; Yu, D.; Ge, C.; Luo, X.; Du, L.; Zhang, X.; Hui, C. Combined effects of microplastics and chlortetracycline on the intestinal barrier, gut microbiota, and antibiotic resistome of Muscovy ducks (Cairina moschata). Science of The Total Environment 2023, 887, 164050.	microplastics, chlortetracycline, co-exposure, animals, toxicity
83. Qiu, X.; Yang, D.; Yu, L.; Song, L.; Yang, L.; Yang, Q. Effect of polyethylene microplastics on tebuconazole bioaccumulation, oxidative stress, and intestinal bacterial community in earthworms. Journal of Hazardous Materials 2024, 480, 136056.	microplastics, antifungals, co-exposure, invertebrates, toxicity
84. Yang, J.; Zheng, C.; Zhang, E.; Smagghe, G.; Gui, S.; Wu, X.; Chen, X. Pyraclostrobin and polyethylene nanoplastics jointly interfere with the antibiotic resistome in earthworm gut. Biology and Fertility of Soils 2025.	microplastics, antifungals, co-exposure, invertebrates, toxicity
85. Bao, X.; Zhou, R.; Cui, Y.; Wang, Z.; Shi, J.; Gao, S.; Wang, X.; Meng, Z.; Chen, X. The carrier effects of aged polyethylene microplastics regulate the toxicological effects of azoxystrobin on earthworms: Interaction relationship of adsorption-desorption behavior and combined toxicity. Environmental Chemistry and Ecotoxicology 2025, 7, 1506-1517.	microplastics, antifungals, combined toxicity, co-exposure, invertebrates, toxicity
86. Sun, W.; Meng, Z.; Li, R.; Zhang, R.; Jia, M.; Yan, S.; Tian, S.; Zhou, Z.; Zhu, W. Joint effects of microplastic and dufulin on bioaccumulation, oxidative stress and metabolic profile of the earthworm (Eisenia fetida). Chemosphere 2021, 263, 128171.	microplastics, antivirals, combined toxicity, co-exposure, invertebrates, toxicity
87. Ma, J.; Sheng, G.D.; O'Connor, P. Microplastics combined with tetracycline in soils facilitate the formation of antibiotic resistance in the Enchytraeus crypticus microbiome. Environmental Pollution 2020, 264, 114689.	microplastics, tetracycline, co-exposure, invertebrates, toxicity
88. Xu, B.; Liu, F.; Brookes, P.C.; Xu, J. The sorption kinetics and isotherms of sulfamethoxazole with polyethylene microplastics. Marine Pollution Bulletin 2018, 131, 191-196.	microplastics, sulfamethoxazole, co-exposure, toxicity
89. Lu, L.; Wan, Z.; Luo, T.; Fu, Z.; Jin, Y. Polystyrene microplastics induce gut microbiota dysbiosis and hepatic lipid metabolism disorder in mice. Science of The Total Environment 2018, 631-632, 449-458.	microplastics, co-exposure, mammals, toxicity
90. Liu, Z.; You, X.-y. Recent progress of microplastic toxicity on human exposure base on in vitro and in vivo studies. Science of The Total Environment 2023, 903, 166766.	microplastics, co-exposure, toxicity
91. Bhagat, J.; Zang, L.; Nishimura, N.; Shimada, Y. Zebrafish: An emerging model to study microplastic and nanoplastic toxicity. Science of The Total Environment 2020, 728, 138707.	microplastics, co-exposure, fish, toxicity

Paper	Keywords
92. Burgos-Aceves, M.A.; Faggio, C.; Betancourt-Lozano, M.; González-Mille, D.J.; Ilizaliturri-Hernández, C.A. Ecotoxicological perspectives of microplastic pollution in amphibians. Journal of Toxicology and Environmental Health, Part B 2022, 25, 405-421.	microplastics, co-exposure, amphibians, toxicity
93. Beggel, S.; Kalis, E.J.J.; Geist, J. Towards harmonized ecotoxicological effect assessment of micro- and nanoplastics in aquatic systems. Environmental Pollution 2025, 366, 125504.	microplastics, co-exposure, aquatic organisms, toxicity
